# G-quadruplexes folding mediates downregulation of PBX1 expression in melanoma

**DOI:** 10.1038/s41392-022-01222-5

**Published:** 2023-01-06

**Authors:** Yutong Sui, Feilin Liu, Song Zheng, Xiaomei Liu, Pingli Sun, Chunli Yao, Yingyao Zhang, Hongwen Gao, Xinghua Gao, Jinyu Liu

**Affiliations:** 1 Department of Toxicology, School of Public Health, Jilin University, Changchun, China; 2 The Second Hospital of Jilin University, Changchun, China; 3 Department of Dermatology, The First Hospital of China Medical University, Key Laboratory of Immunodermatology, Ministry of Education/Public Health, Shenyang, China

**Keywords:** Cancer therapy, Skin cancer

**Dear Editor**,

Pre-B-cell leukemia homeobox transcription factor 1 (PBX1) is identified at t(1;19)chromosomal translocations in acute pre-B-cell leukemia and is involved in regulating multiple biological processes.^1^ Importantly, accumulating evidence have suggested that dysregulation of PBX1 have been shown to be involved in tumorigenesis, poor prognosis and drug resistance.^1^ It has been shown that the expression of PBX1 is increased in melanoma cells and overexpression of PBX1 significantly promotes the melanoma cell growth.^2^ However, the clinical impact of PBX1 on melanoma and the molecular mechanisms regulating PBX1 expression in melanoma are largely unknown.

We first investigated PBX1 expression in human melanoma tissues. We first detected the PBX1 expression in a cohort (Cohort 1, Supplementary Table 1) containing 47 pairs of melanoma tissues and adjacent non-cancerous tissues by performing immunofluorescence (IF) assays. We noticed that PBX1 is highly expressed in melanoma tumors, especially in advanced tumors (Fig. 1a, b and Supplementary Fig. 1a–d). We further detected the PBX1 expression in another cohort (Cohort 2, Supplementary Table 2) containing 48 melanoma tissues and 15 normal tissues by performing immunohistochemistry (IHC) assays. The PBX1 expression was also higher in melanoma tissues of Cohort 2 (Fig. 1c, d). Unexpectedly, PBX1 RNA levels were lower in melanoma tissues of The Cancer Genome Atlas (TCGA) database (Supplementary Fig. 1e), which is inconsistent with our results (Supplementary note). Kaplan–Meier analysis of Cohort 2 and TCGA revealed that high PBX1 expression in melanoma tissues correlates with reduced overall survival and metastasis-free survival (Fig. 1e, f and Supplementary Fig. 1f). We also found that overexpression of PBX1 significantly promotes the proliferation ability of mouse primary melanocytes, while PBX1 knockout inhibits the proliferation, migration and invasion of B16-F10 cells (Supplementary Fig. 1g–m). Furthermore, we found that PBX1 can activate the NF-κB pathway (a well-known oncogene pathway involved in melanoma tumorigenesis and progression) by promoting the nuclear translocation of NF-κB in melanoma (Supplementary Figs. 2a–d, 3a–c and Supplementary Table 3). Collectively, these results demonstrate that PBX1 functions as an oncogene in melanoma and its high expression predicts poor prognosis of patients with melanoma.

**Fig. 1:**
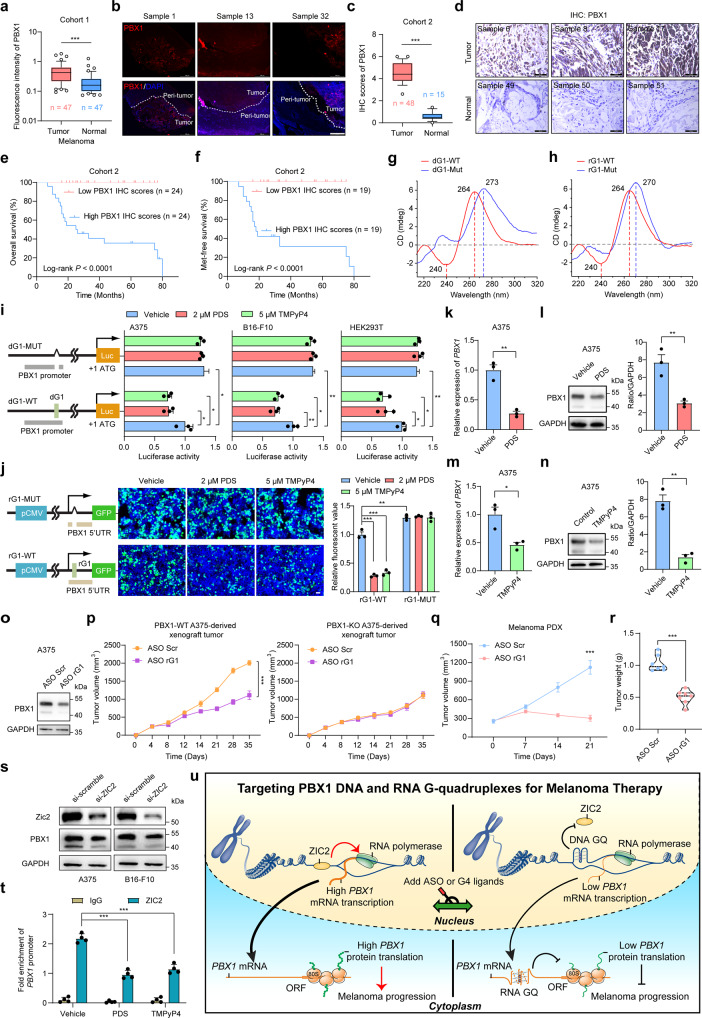
PBX1 G4s formation inhibits melanoma progression by downregulating PBX1 expression. **a** PBX1 expression was quantified by immunofluorescence (IF) assays in tumor and non-tumor tissues of patients from the melanoma cohort 1. **b** The representative images of PBX1 IF intensity in melanoma tissues and adjacent non-cancerous tissues. Scale bar, 500 μm. **c** PBX1 expression was quantified by immunohistochemistry (IHC) assays in tumor and non-tumor tissues of patients from the melanoma cohort 2. **d** The representative images of PBX1 IHC staining in HCC tissues and noncancerous skin tissues. Scale bar, 50 μm. **e**, **f** The overall (**e**) and metastasis-free (Met-free) survival (**f**) analyses for patients from the melanoma cohort 2. The high (blue) and low (red) expression of PBX1 in melanoma tissues were determined by the median of PBX1 expression levels in all melanoma samples or the melanoma samples without metastasis. Survival curves were calculated using Kaplan–Meier method, and the significance was determined by log-rank test. **g**, **h** CD spectrum of WT and Mut dG1 (**g**). CD spectrum of WT and Mut rG1 (**h**). CD, circular dichroism. **i** The activity of G-quadruplex in HEK293T, A375 and B16-F10 cells were performed by luciferase reporter assays. **j** HEK293T, A375 and B16-F10 cells were treated with or without 2 μM PDS or 5 μM TMPyP4 for 48 h. Left: representative confocal images. Scale bars: 100 μm. Right: the relative fluorescent value was measured. **k**–**n** The mRNA and protein levels of PBX1 in A375 cells were detected by qRT-PCR and western blot. PDS (2 μM) or TMPyP4 (5 μM) treatment for 48 h inhibits the PBX1 mRNA (**k**, **m**) and protein levels (**l**, **n**. From left to right: PBX1 protein level, statistical analysis of western blot results. PBX1 protein levels were normalized to the GAPDH protein levels.). **o** The protein levels of PBX1 in A375 with ASO Scr or ASO rG1 (25 nM) treatment for 48 h. **p** PBX1-wild type (WT) or PBX1-knockout (KO) A375 cells-derived xenograft tumor volume measurements at day 0, 4, 8, 12, 14, 21, 28 and 35. **q** Melanoma patient-derived xenograft (PDX) tumor volume measurements at day 0, 7, 14 and 21. (*n* = 7 mice/group). **r** Melanoma PDX tumor weight of mice neoplasm after mice sacrifice. **s** Relative expression levels of ZIC2 and PBX1 expressing si-scramble or si-ZIC2, detected by western blot. **t** The occupancy of ZIC2 in the promoter region of PBX1 was measured by ZIC2 ChIP in A375 cells with or without PDS and TMPyP4 treatment, followed by qRT-PCR. **u** The schematic of targeting PBX1 G-quadruplexes for melanoma therapy (Figure was modified from Servier Medical Art (http://smart.servier.com/), licensed under a Creative Common Attribution 3.0 Generic License. (https://creativecommons.org/licenses/by/3.0/)). Data are shown as mean ± SEM of three independent experiments, two-tailed Student’s *t* test. SEM standard error of mean. *P < 0.05, **P < 0.01, ***P < 0.001

G-quadruplexes (G4s) are 4-stranded structures formed in guanine (G)-rich DNA or RNA strands and have been shown to involve in tumorigenesis by regulating the oncogene expression.^3,4^ Worth to note, PBX1 promoter and transcript are extremely rich in guanines, suggesting that G4s may form in PBX1 promoter and transcript. Thus, we determined to screen potential G4s in the promoter and transcript of PBX1 (Supplementary Fig. 4a). By combing 2 independent G4 prediction software, we identified 2 potential DNA G4s (named as dG1 and dG2) in PBX1 promoter region and 3 potential RNA G4s (named as rG1, rG2 and rG3) in the transcript of PBX1 (Supplementary Table 4). Due to the high conservation and G4 folding capabilities of dG1 and rG1 (Supplementary Fig. 4b, c), we chose them as typical targets for further study. Furthermore, we confirmed the formation of dG1 and rG1 both in vitro and in live cells by adopting several assays (Fig. 1g, h, Supplementary Fig. 5a–e and Supplementary Table 5). Meanwhile, we found that G4-specific ligands pyridostatin (PDS) and 5,10,15,20-tetrakis-(Nmethyl-4-pyridyl) porphine (TMPyP4) can bind and stabilize dG1 and rG1 (Supplementary Fig. 5f–i and Supplementary Table 6).

Given that G4s in the promoter and transcript contribute to regulate transcription and translation associated processes,^3^ we next investigated the effects of dG1 and rG1 on PBX1 transcription and translation. By performing luciferase and enhanced green fluorescent protein (EGFP) reporter assays, we found that dG1 and rG1 formation induced by PDS and TMPyP4 treatment can inhibit the PBX1 transcription and translation respectively (Fig. 1i, j). As expected, dG1 and rG1 formation significantly decreased the mRNA and protein levels of PBX1 in A375 and B16-F10 melanoma cells (Fig. 1k–n and Supplementary Fig. 6a–d). In addition, we investigated whether PBX1 G4s formation inhibits melanoma progression by downregulating PBX1 expression. We found that PDS and TMPyP4 treatment significantly inhibit the proliferation, colony formation, migration and invasion of A375 and B16-F10 cells (Supplementary Fig. 7a–h). Consistent with in vitro results, treatment of PDS and TMPyP4 significantly inhibits the xenograft tumor growth and metastasis in vivo by downregulating PBX1 expression (Supplementary Figs. 8a–i, 9a–l). Taken together, these results indicate that PBX1 G4s formation induced by PDS and TMPyP4 suppresses melanoma progression by downregulating PBX1.

Although PDS and TMPyP4 showed potent anti-tumor effect on melanoma, their anti-tumor effects may be due in part to its binding to other cellular G4s and the off-target effect may also cause side effects and cytotoxicity, limiting their clinical applied potentials for human disease therapy. Antisense oligonucleotides (ASOs) show promising clinical applied potentials and have been used to induce specific RNA G4s formation.^5^ Thus, we designed the ASO specifically targeting rG1 complementary sequences in PBX1 5’ UTR to induce PBX1 rG1 formation (Supplementary Fig. 10 and Supplementary Table 7). Indeed, ASO treatment could increase the pull-downed PBX1 transcripts by G4-specific antibody BG4 (Supplementary Fig. 11a), suggesting the induction of rG1 formation by ASO. And we found that PBX1 rG1 formation induced by ASO significantly suppresses melanoma progression and the NF-κB pathway in A375 cells, patient-derived xenograft (PDX)-derived tumor cells (PDCs) and patient-derived xenograft (PDX) mouse models (Fig. 1o–r and Supplementary Fig. 11b–i). Furthermore, we found that ASO treatment shows no significant effect on the growth of stable PBX1-knockout A375 cells both in vitro and in vivo (Fig. 1o, p and Supplementary Fig. 12a–d), suggesting that ASO inhibits melanoma growth in a PBX1 dependent manner. More importantly, PBX1-overexpressed melanoma cells were more sensitive to G4 ligands (PDS and TMPyP4) and ASO (Supplementary Fig. 13a, b).

Differently from G4s located 5’ UTR of mRNA, which impede mRNA translation via steric blocking effects, G4s within promoter region regulate transcription by recruiting or blocking the occupancy of transcriptional factors in promoter regions.^3^ Thus, we next investigated the transcriptional factors involved in the regulation of dG1 on PBX1 transcription. By using transcriptional factors prediction software PROMO, nine transcriptional factors were predicted to bind to the dG1 site in PBX1 promoter (Supplementary Fig. 14a). Among them, we found that the expression of ZIC1, ZIC2 and ZIC3 positively correlate with the PBX1 expression in melanoma respectively (Supplementary Fig. 14b), and chromatin immunoprecipitation (ChIP) assays showed the most remarkable enrichment of ZIC2 in PBX1 promoter regions (Supplementary Fig. 14c). Indeed, knockdown of ZIC2 inhibited the expression of PBX1; whereas overexpression of ZIC2 had completely opposite effects (Fig. 1s and Supplementary Fig. 14d–f). Worth to note, PDS or TMPyP4 treatment caused a significant reduction in the strength of ZIC2 binding at the promoter of PBX1 (Fig. 1t and Supplementary Fig. 14g), suggesting the inhibitory effect of dG1 formation on the binding of ZIC1 to PBX1 promoter region. Taken together, PBX1 dG1 formation decreased the expression of PBX1 by blocking ZIC2 occupancy in PBX1 promoter regions.

In summary, we had shown that PBX1 is up-regulated in melanoma and its high expression predicts poor prognosis of patients with melanoma. In addition, we proved the presence of G4s motifs in the promoter and 5’ UTR of PBX1. PBX1 G4s formation induced by G4 stabilizing compounds TMPyP4 and PDS significantly inhibited the melanoma growth and metastasis in vitro and in vivo by downregulating the PBX1 expression. However, due to the low specificity and druggability, TMPyP4 and PDS could not be used as drugs and were usually employed as a tool to study the function of G4s in cancer or other diseases. Thus, we designed specific ASO targeting rG1 in 5’ UTR of PBX1 and confirmed that PBX1 rG1formation induced by ASO can significantly inhibit melanoma progression. Mechanistically, PBX1 DNA G4 formation blocks ZIC2 occupancy in PBX1 promoter regions to inhibit the transcription of PBX1, while PBX1 RNA G4 inhibits the translation of PBX1 via steric blocking effects (Fig. 1u). Our findings collectively revealed the clinical impact of PBX1 on melanoma and a novel regulatory mechanism for governing PBX1 expression and provided another therapeutic target that can combine with existing therapeutic strategy against melanoma or other cancers.

## Supplementary information

Supplementary Information

Supplementary Table 3

## Data Availability

All data reported in this paper will be shared by the lead contact upon request. The data of transcriptome sequencing were submitted to the SRA database. The accession number is PRJNA865294. Any other information required to reanalyze the data reported in this paper is available upon request.
