# Supplementary material for: G-quadruplexes folding mediates downregulation of PBX1 expression in melanoma
Source: Signal Transduct Target Ther. 2023 Jan 6;8:12. doi: 10.1038/s41392-022-01222-5 (PMC9816092; doi:10.1038/s41392-022-01222-5)
Supplement: Supplementary file 1 — Supplementary Information [file 41392_2022_1222_MOESM1_ESM.docx]

Supplementary Materials for

G-quadruplexes folding mediates downregulation of PBX1 expression in melanoma

Yutong Sui^1^, Feilin Liu^2^, Song Zheng^3^, Xiaomei Liu^1^, Pingli Sun^2^, Chunli Yao^2^, Yingyao Zhang^1^, Hongwen Gao^2*^, Xinghua Gao^3*^, Jinyu Liu^1*^

^1^Department of Toxicology, School of Public Health, Jilin University, Changchun, China.

^2^The Second Hospital of Jilin University, Changchun, China.

^3^Department of Dermatology, The First Hospital of China Medical University. Key Laboratory of Immunodermatology, Ministry of Education/Public Health, Shenyang, China. Centre for Theranostics of Immnological Skin Diseases, Committee of Development and Reform, China

Correspondence to: [gaohongwen@jlu.edu.cn](mailto:gaohongwen@jlu.edu.cn)

[jy_liu@jlu.edu.cn](mailto:jy_liu@jlu.edu.cn)

**This PDF file includes:**

- Supplementary Notes
- Supplementary Methods
- Supplementary References
- Supplementary Tables 1-9
- Supplementary Figures 1-13

**Supplementary Note. The expression levels of PBX1 in melanoma tissues**

By using Gene Expression Profiling Interactive Analysis (GEPIA, <http://gepia.cancer-pku.cn>), we had examined the expression levels of PBX1 in melanoma cohort from TCGA data. We observed that the mRNA levels of PBX1 are higher in normal tissues than in tumor tissues (**Supplementary Fig. 1e**), inconsistent with our data of Cohort 1 and Cohort 2 (**Fig. 1a-d**) and those in previous reports[^1^](#_ENREF_1). The clinical data of PBX1 from TCGA indicated that PBX1 may act as a tumor suppressor in melanoma. However, our in vitro and in vivo results (**Supplementary Fig. 1g-m**) and prior research[^2^](#_ENREF_2) suggested the potential oncogene role of PBX1. It seems to be contradictory. There are several probable reasons given for that including: (1) due to the lack of paracancer tissue in TCGA databases, the normal controls from the GTEx database are used, which may lead to inaccurate differential expression data of PBX1; (2) The expression of PBX1 in TCGA melanoma cohort is RNA levels, while the expression of PBX1 in our cohort 1 and cohort 2 were protein levels. Due to the cellular unknown regulatory mechanism, the RNA and protein expression of PBX1 in melanoma may be inconsistent; (3) the causal relationship between the analysis of TCGA data and the function of target genes is not absolutely precise, the function of many well-known genes and the analysis of TCGA data is contradictory. For example, it is widely reported that PBX1 play oncogene roles in breast cancer. However, the PBX1 expression in breast tissues from TCGA database exhibited no significant change compared with that in normal tissues, which is inconsistent with the oncogene roles of PBX1 in breast cancer. Thus, the real expression of PBX1 in melanoma deserves in-depth study in the future.

**Supplementary Methods**

**Clinical Specimens**

The study included 110 melanoma patients was divided into two independent cohorts. Cohort 1: 47 melanoma samples and adjacent noncancerous tissues who initially underwent surgery and were diagnosed with melanoma were collected from The Second Hospital of Jilin University between September 2018 and December 2020 (Changchun, China). All patients were categorized by gender, age at diagnosis, and primary tumor localization. Additionally, we collected relevant primary tumor parameters, including Breslow thickness, ulceration, Clark level and Tumor-Infiltrating Lymphocytes. The patients in cohort 1 had no follow-up information and were only used for detecting the expression of PBX1. Cohort 2: We randomly selected 63 (38 primary melanoma, 10 metastatic melanomas in lymph node, 7 normal skin tissue, 2 mucosa of pharynx, 2 esophageal mucosa tissue, 2 Small intestinal mucosa, 2 lymph node tissue) melanoma formalin-fixed, paraffin-embedded tissues from The Second Hospital of Jilin University between January 2012 and July 2018. The patients in cohort 2 had complete follow-up information and were used for detecting the expression of PBX1 and survival analyses. The collected information of clinicopathological parameters of cohort 1 and cohort 2 were unmatched, thus the cohort 1 and cohort 2 were not be analysed all together.

All tumor tissue from each case was formalin-fixed and paraffin-embedded for routine diagnosis. This protocol was approved by the Ethical and Scientific Committees of The Second Hospital of Jilin University, and all patients provided written informed consent before tissue collection. Detailed patients’ clinical information is listed in **Supplementary Table 1** and **Supplementary Table 2**.

**Cell culture, treatment, murine primary melanocytes isolation and proliferation assays**

The human melanoma cell line A375, mouse melanoma cell line B16-F10 and human embryonic kidney cell line HEK293T were obtained from the China Center for Type Culture Collection (CCTCC, Wuhan city, China). All the cell lines used in this paper were grown in Dulbecco’s modified Eagle’s medium (DMEM) (Gibco, Thermo Fisher, Waltham, MA), supplemented with 5% fetal bovine serum (Gibco, Thermo Fisher, Waltham, MA) and 2 mM L-glutamine (Gibco, Thermo Fisher, Waltham, MA) at 37℃ with 5% CO_2_. Murine primary melanocytes isolation was developed previously[^3^](#_ENREF_3) and cultured in modified Ham’s F12 medium (Gibco, Thermo Fisher, Waltham, MA). To assess cell proliferation, melanocytes were cultured in melanocyte medium with or without phorbol 12-myristate 13-acetate (PMA)[^4^](#_ENREF_4)^,^ [^5^](#_ENREF_5), then fixed with 4% w/v paraformaldehyde and stained with DAPI nuclear stain at various times and counting the cells at various times[^5^](#_ENREF_5).

**UID mRNA Sequencing and Data Analysis**

The stable PBX1-overexpressed and control A375 cells were used for UID mRNA sequence. The total RNA was extracted, amplified, transcribed and then sequenced on Illumina sequencer with the help of Seqhealth Technology Co., LTD (Wuhan, China Catalog NO. 2022-G0385). Distinctively expressed genes were used to draw heat maps and conduct the GO, KEGG and GSEA ontology enrichment analysis. Genes with fold change [FC] ≥ 2 and P < 0.05 (Student’s *t* test) were recruited as differentially expressed genes. Gene set enrichment analysis (GSEA) was performed in PBX1_OE vs. NC A375 cells based on the Molecular Signatures Database (MSigDB) gene set collection (v6.0).

**G-quadruplexes and transcriptional factors analysis**

The complete genome and transcript sequences of PBX1 were retrieved from the NCBI Genome database (https://www.ncbi.nlm.nih.gov/genomes). The FASTA sequences of the complete genome and transcript sequences of PBX1 were then used for the prediction of the putative G4-forming sequences (PQSs) with QGRS-mapper[^6^](#_ENREF_6) and Pqsfinder^[7](#_ENREF_7)^.

The QGRS-mapper (<https://bioinformatics.ramapo.edu/QGRS/analyze.php>) and Pqsfinder (https://pqsfinder.fi.muni.cz/) was run using their online tools.

For evaluating the G4 folding capabilities by the consecutive G over consecutive C ratio (cGcC), G4Hunter (G4H) and G4 neural network (G4NN) scores, the G4RNA screener (http://scottgroup.med.usherbrooke.ca/G4RNA_screener/) were used[^8^](#_ENREF_8).

cGcC was implemented to address the issue of competition in between G4 and Watson-Crick based structures. The presence of cytosine runs in the vicinity of a potential G4 was demonstrated to be an important feature to consider in the identification of potential G4 since the base pairing of those C runs with G runs involved in the potential G4 can hinder its formation. This score varies on a logarithmic scale by its ratio nature[^9^](#_ENREF_9). In this study, PQSs with a ratio value superior to the arbitrary threshold of 4.5 were considered as being accessible under conditions favouring G4 folding.

G4H was designed in a similar way to the cGcC score but was built to analyze DNA sequences. We demonstrated its relevance in RNA as well. It attributes an increasing positive score to each contiguous G and a negative counterpart for contiguous C. The sequence is then scored by the average of the values[^10^](#_ENREF_10). In this study, PQSs with a ratio value superior to the arbitrary threshold of 0.9 were considered as being accessible under conditions favouring G4 folding.

Sequences of the G4RNA database were converted into vectors of their trinucleotide content to train an artificial neural network. This artificial neural network, G4NN, evaluates the similarity of a given sequence to known G-quadruplexes and reports it as a score between 0 and 1[^11^](#_ENREF_11). In this study, PQSs with a ratio value superior to the arbitrary threshold of 0.9 were considered as being accessible under conditions favouring G4 folding.

For the analysis of the conservation of the PBX1 dG1 sites throughout 100 species, the results were retrieved from the University of California Santa Cruz (UCSC) genome browser.

For the analysis of transcriptional factors around the PBX1 dG1 genomic sites, we used the online software PROMO[^12^](#_ENREF_12), which a set of tools to construct positional weight matrices from known transcription factor binding sites in a species or taxon-specific manner, and to search for matches in DNA sequences.

**Fluorescence assays**

For NMM fluorescence assays, the DNA or RNA oligonucleotide samples pre-heated at 95 °C for 10 min were slowly cooled to room temperature before co-incubating with NMM. Then, 0.6 μM NMM and 0.3 μM samples were co-incubated 4 hours. The NMM fluorescence assays were performed by using JASCO FP-6500 spectrofluorometer at room temperature.

**Circular dichroism (CD) measurements**

For CD spectra and CD melting experiments, the DNA or RNA oligonucleotide samples pre-heated at 95 °C for 10 min were slowly cooled to room temperature, and then were incubated at 4 °C for 24 h. The samples were detected by JASCO J-810 spectropolarimeter equipped with a temperature-controlled water bath. In CD melting experiments, versus temperature were collected at a heating rate of 1 °C min^−1^.

**Nondenaturing polyacrylamide gel electrophoresis experiments**

Native gel electrophoresis was performed by acrylamide gel (15 %) and run at room temperature, 1 × TB buffer containing 10 mM KCl and was silver stained. About 2 μM RNAs were loaded on the gel.

**Oligonucleotides and antisense oligonucleotides**

All DNA and RNA oligonucleotides were synthesized by Shanghai Sangon Biological Engineering Technology & Services (Shanghai, China) and the antisense oligonucleotides were synthesized by Tsingke Biotechnology Co., Ltd (Beijing, China). The oligonucleotide sequences were described in **Supplementary Table 9**. Concentrations of the oligomers were determined by NanoDrop Lite Spectrophotometer (Thermo, USA).

**Chemicals, plasmids, bacterial strains and antibodies**

TMPyP4 (Selleck, USA; Cat. #P1202) and Pyridostatin (PDS; Selleck, USA; Cat. #S7444) was dissolved and stored at −80°C. Both TMPyP4 and PDS were diluted into desired working concentrations in culture medium prior to experiments. All chemicals were used without further purification. pLVX-IRES-mcherry (Cat. #VT1461), PBX1 promoter pGL3-Basic (Cat. #VT15541) and pFLAG-CMV 2 (Cat. #VT1068) were from YouBio (China). Anti-GAPDH (Cat. #ab9485), anti-ZIC1 (Cat. #ab134951), anti-ZIC2 (Cat. #ab150404) and anti-ZIC3 (Cat. #ab189969), anti-NF-κB p65 (Cat. #ab32536), anti-Histone H3(Cat. #ab1791), Human IL-6 ELISA Kit (Cat. #ab178013), Mouse IL-6 ELISA Kit (Cat. #ab222503), Human MIP2 (CXCL2) ELISA Kit (Cat. #ab184862) and Mouse MIP2 (CXCL2) ELISA Kit (Cat. #ab204517) were from Abcam (UK). Anti-PBX1 (Cat. #4342S) and anti-FLAG (Cat. #14793S) were from CST (USA). Anti-PBX1 (Cat. # PA5-82118) and phorbol 12-myristate 13-acetate (PMA) (Cat. #00-4970-03) were from Invitrogen (USA). Anti-BG4 (Cat. #Ab00174-30.126) was from Absolute Antibody (UK).

**Mice**

All of the 4–6 weeks specific pathogen free (SPF) C57BL/6 and male BALB/C nude mice used in this study were purchased from Beijing Huafukang Experimental Animal Technology Co., Ltd (Beijing, China). NCG (Cat. #T001475) mice was from GemPharmatech (Jiangsu, China). All mice were raised under specific pathogen-free conditions for 2 weeks before experiment. All *in vivo* experiments were approved and supervised by the Institutional Animal Care and Use Committee of School of Public Health Jilin University (Changchun, China).

**Immunofluorescence assay**

Melanoma tumor tissues were paraffin-embedded for analysis. Samples were incubated with primary antibody for 12 hours at 4°C. Secondary antibodies were conjugated with Alexa594-labeled secondary antibody (Invitrogen, USA) for 2 hours at 37°C. 4',6-diamidino-2-phenylindole (DAPI) was added in the dark before taken images with a microscope (Leica, [Germany](javascript:;)), and the statistical analysis was performed by Image J. Six micrographs per field were performed.

**Immunohistochemistry (IHC) assay**

Formalin-fixed, paraffin-embedded melanoma tumors, liver, spleen, lung and renal were trimmed and processed into paraffin-embedded tissue blocks. Tissue sections cut from the blocks for immunohistochemistry were mounted onto glass slides, deparaffinized in xylene, and rehydrated in ethanol. Pretreatment by Trypsin (0.1%), then add Peroxidazed blocking reagent for 5 min. Blocked slides were incubated antibodies. The slides were then incubated with a rabbit-on-canine horseradish peroxidase secondary antibody. NanoZoomer 2.0-RS Digital Pathology (Hamamatsu, Japan) was used to taken images. Six micrographs per field were performed.

**Overexpression of *PBX1* and *ZIC2***

To assessment the effects of PBX1 overexpression on melanoma, we used *PBX1*-overexpressed B16-F10 cell lines and murine primary melanocytes. For *PBX1* overexpression, the *PBX1* CDS sequences were cloned into pLVX-IRES vector. The constructs were conﬁrmed by DNA sequencing. The vectors with the CDS sequence of *PBX1* can be transfected into B16-F10 cells and melanocytes using Lipofectamine 2000 (Invitrogen, USA), following the manufacturer’s instructions. The vectors also can be used to construct the stable *PBX1*-overexpressed B16-F10 cell lines and murine primary melanocytes. Briefly, the vectors along with viral packaging plasmids (pMD2.G and psPAX2) were transfected into human embryonic kidney 293T cells using Lipofectamine 2000 (Invitrogen, USA), following the manufacturer’s instructions. Virus supernatant was harvested after 48 h, filtered through a 0.45 μM filter, and incubated on target cells for 6 h at a 1:10 dilution with 8 μg/mL polybrene. Infected cells were selected with 200 μg/mL puromycin for 2 weeks before evaluation for knockdown efficiency. All the media contained 10% fetal bovine serum (FBS), 100 units/mL penicillin, and 100 mg/mL streptomycin (Gibco, USA). Cells were incubated in humidified incubators equilibrated with 5% CO2 at 37 °C.

For *ZIC2* overexpression, the human *ZIC2* and mouse *Zic2* CDS sequences were cloned into pFLAG-CMV 2 vectors. The constructs were conﬁrmed by DNA sequencing. The vectors with the CDS sequenceswere transfected into B16-F10 and A375 cells respectively using Lipofectamine 2000 (Invitrogen, USA), following the manufacturer’s instructions.

**PBX1 knockout**

To construct PBX1 knockdown A375 or B16-F10 cell line, a single guide RNA (sgRNA) for a frameshift mutation of the PBX1 (sequence: TGTCCCAGCACTTGCAGGAT) was designed by Crispr Direct (http://Crispr.dbcls.jp). Annealed double-stranded DNA was inserted into the px458 vector using the BbsI (NEB, USA) site. Then the purified recombinant plasmid was transfected into A375 or B16-F10 cells using Lipofectamine 3000 (Invitrogen). After puromycin screening, cells were separated into 96-well plates by limiting dilution and collected to test the knockout efficiency. The resulting screened, stable PBX1 knockout cell line was used for phenotypic and tumor experiments *in vivo*.

**RNA Extraction and quantitative RT-PCR assay**

Total RNAs were extracted from cultured cells using TRIzol (Invitrogen, USA) and then converted to cDNAs using the SuperScript III First Strand Synthesis System (Invitrogen, USA). The qRT-PCR assays were performed in the ABI 7500 (Invitrogen, USA) using SYBR Green^®^ Mix (Invitrogen, USA). The relative expression of RNAs was calculated using the comparative Ct method. The primer sequences were described in **Supplementary Table 9**.

**Western blot assay**

For protein analysis of whole-cell lysates, cells were lysed in RIPA (CWBIO, Beijing, China) buffer. For tumors, liquid nitrogen was used to ground tissues first. Then, lysates were generated using RIPA (CWBIO, Beijing, China) buffer on ice for 20 min, followed by centrifugation for 15 min at 13,000 rpm at 4°C. Protein-containing supernatant was transferred to fresh microcentrifuge tubes and stored at −20℃ (Short-term preservation) for next step. *BIO-RAD* DC Protein Assay (*BIO-RAD*, USA) was performed to quantify the protein. Total proteins were electrophoresed on SDS-polyacrylamide gels. Then, proteins were transferred to polyvinylidene difluoride (PVDF) membrane.

Membranes were washed with TBS-T (Invitrogen, USA) for 6 times (5 min for every time) after incubation with primary antibodies on a plate shaker overnight at 4°C. Then, the blots were incubated with secondary antibodies conjugated to horseradish peroxidase (HRP) for 60 min at RT on a plate shaker. Membranes were washed with TBS-T (6 × 5 min).  The immunoreactive bands were detected using SuperSignal™ West Pico chemiluminescent substrate kit (Thermo Fisher Scientific, USA) and Western blot detection system (BioRad, USA). The GAPDH was used as a loading control for western blot assays. Band intensity levels were normalized to GAPDH.

**Enzyme linked immunosorbent assay (****ELISA)**

Cells were plated at ~80% confluency. Twenty-four hours later, cells were washed three times with PBS and cultured in serum-free medium, and 48 h later, cells were collected and analyzed in duplicate with a human IL6 or CXCL2 ELISA Kit.

**Colony formation assay**

For colony formation assays, A375 and B16-F10 cells pretreated with 2 μM PDS or 5 μM TMPyP4 for 24 h were seeded at a density of 500-1000 cells in 12-well plates. After 2 weeks, the colonies were stained with 1% crystal violet at room temperature and washed in PBS and tap water, then counted the colony numbers.

***In vitro* cell invasion assay**

Transwell invasion assay was used Matrigel as the intervening invasive barrier in upper chamber membrane (Corning, USA). The lower transwell chamber was filled with 2.5% serum containing DMEM medium. Cells were suspended in upper chamber. After incubation for 24 h, the filters were removed, and the cells on the membrane were fixed with methanol. Invasion of cells into surrounding matrix were stained with 0.5% crystal violet. The dye was washed with water, and the cells were examined by microscopy (Leica, Germany). Six micrographs per individual were performed.

**Design of antisense oligonucleotides (ASOs) targeting PBX1 RNA G4s**

RNA G4 structure is a motif that is inherently part of a greater RNA context, the folding of which may be influenced by neighboring sequences. The structure of RNA G-rich sequence may dynamically transform between G4 and stem loop structures, thus targeting the G-rich complementary sequence by ASOs would promote RNA G4 formation. The ASO, which can induce the formation of PBX1 rG1 formation, should bind to the complementary sequence of rG1 in the 5’UTR of PBX1 mRNA. Thus the RNA secondary structure of the 5’UTR of PBX1 mRNA was predicted and the complementary sequence of rG1 was identified (**Supplementary Fig. 10**). The ASOs targeting the rG1 complementary sequence were synthesized by Tsingke Biotechnology Co., Ltd (Beijing, China). The ASO sequences were described in **Supplementary Table 9**.

***In vivo* tumor growth assays**

To assess the effects of PBX1 overexpression on tumor growth *in vivo*, six-week-old male athymic nude mice (BALB/C background) were randomly divided into indicated groups (5 mice/group) before inoculation. 1 × 10^6^ B16-F10 melanoma cells transfected with pLVX-Ctrl or pLVX-PBX1 were subcutaneously injected into the upper flank of the mice back. After injection, the neoplasm weight was measured after sacrifice.

To determine the effects of PDS and TMPyP4 on tumor growth *in vivo*, 6-week-old C57/BL6 mice and male BALB/C mice were implanted into the upper flank by subcutaneously injection with 1 × 10^6^ B16-F10 and A375 melanoma cells. When the tumors reached a size of 400–450 mm^3^ and the mice were randomized into indicated groups (5 mice in each group) and injected intraperitoneally with the TMPyP4 (20 mg/Kg; 30 mg/Kg) and PDS (5 mg/Kg) (Three TMPyP4 treatments have been carried out at days 3, 6, 9, 12, 15, 18 and 21; PDS administration twice a day for 21 days.).  At day 21,12 h post the injection, mice were sacrificed, the tumor were used to perform the qRT-PCR, IHC assay and the liver, spleen, lung and renal were collected for further analysis.

To determine the effects of ASO rG1 on PBX1 knockout A375 cells-derived xenograft tumor growth *in vivo*, 6-week-old male BALB/C mice were implanted into the upper flank by subcutaneously injection with 2 × 10^6^ B16-F10 and A375 melanoma cells. When the tumors reached a size of 400–450 mm^3^ and the mice were randomized into indicated groups (5 mice in each group) and treated with ASO rG1 (i.v., 20 mg/kg).  At day 35, mice were sacrificed, the tumor were used to perform the qRT-PCR, IHC assay.

**Dual‑ Luciferase reporter assay**

For PBX1 luciferase reporter assay, the luciferase reporter gene plasmids for PBX1 and pRL-TK plasmid were co-transfected into HEK293T, A375 and B16-F10 cells by Lipofectamine 2000 (Invitrogen, USA) in 96-well plates. Forty-eight hr after transfection, cells were harvested and analyzed using Dual-Luciferase® Reporter Assay System (Promega, USA). Firefly activity was normalized to Renilla luciferase activity.

**EGFP reporter vectors construction**

A reporter vector with the pLV-EGFP-N backbone (Inovogen Tech. Co., Beijing, China) was generated to encode the PBX1 5’ UTR sequences with rG1 site. By using the Strata gene Quik Change Site-Directed Mutagenesis kit, we point mutated the rG1 site.

**Cell proliferation assay**

Cell viability was evaluated by Cell Counting Kit-8 (CCK-8) assay according to the manufacturer’s instructions (Abcam, UK). Cells (2000 cells per well) were plated in 96-well plates in triplicate then added CCK-8 to each well at a final concentration of 10% at different time points, and incubation continued at 37°C. After 60 min, the absorbance of the samples was measured at 450 nm. The data were analysed using GraphPad Prism software.

**The scratch-wound assay**

The cells were seeded in a 12-well plate and grown for 24 h to 80% confluence. A denuded area was created across the diameter of the dish by a tip. After treatment with the PDS (2μM) and TMPyP4 (5μM) for 24 h, the cells were washed with PBS and taken pictures with a microscope (Leica, Germany) to evaluate cell migration. Six micrographs per individual were performed.

***In vivo* tumor metastasis assays**

Tail-vein tumor injections were administered with 2 × 10^5^ B16-F10 cells into the lateral tail vein. Metastatic end-point was determined per experiment by maximum primary tumor burden and/or the appearance of moribund mice within all treatment groups as defined within protocols established by the NCI Animal Care and Use Committee. Administration with PDS (5mg/kg) for 30 days (twice a day, *via* i.p.).

**Bioluminescent tumor cell tracking**

For *in vivo* whole-animal imaging, anesthetized mice received 3 mg of D-luciferin (MCE, USA) *via* intraperitoneal injection (i.p.). Luminescence readings were collected on a IVIS system (Perkin Elmer, USA). For *ex vivo* tissue imaging, tissues were perfused with PBS, harvested, and incubated in 1 µg/ml−1 D-luciferin in PBS. Data analysis was conducted using the IVIS Image software package.

**Histopathology assay**

The liver, lung, spleen and kidney were used to examine the histopathological changes of mice. These tissues were fixed in the 4% formalin (BBI Life Sciences Corporation, China). After embedding in paraffin, tissue sections were cuted and stained with Hematoxylin and eosin (HE. Applygen Technologies Inc., China). Six micrographs per individual were performed. Sections were examined with NanoZoomer 2.0-RS Digital Pathology (Hamamatsu, Japan).

**PDX model of melanoma**

Fresh tumor tissue (Detailed message in **Supplementary Table 8** ) was collected after surgery immediately and put it in transport medium consisting of RPMI 1640 medium supplemented with penicillin/streptomycin (100 U/ml; 100 μg/ml), fungizone (1 μg/ml), and gentamicin (50 μg/ml) (all from Gibco, USA)[^13^](#_ENREF_13)^,^ [^14^](#_ENREF_14) for xenotransplantation. Fresh tumor samples from melanoma patients were collected following informed consent for engraftment in accordance with the ethical guidelines approved for this study by the Institutional Review and the Ethics Committee of The Second Hospital of Jilin University. Mice were maintained and all procedures were performed under the approval and supervision of the Institutional Animal Care and Use Committee (IACUC) of Public Health Jilin University (Changchun, China).

Necrotic tissues were removed before transplantation and the tumor tissue was minced into pieces measuring 2 × 2 × 2 mm^3^, and implanted subcutaneously into the flank region of 6-weeks old NCG male mice to generate the first-generation (F1) PDX mice. Successfully engrafted tumor models were then passaged and banked using standard methods. H&E stain was used to evaluate the morphology from patient tumor samples and xenografts of established PDX models.

Treatment with ASO Scr or ASO rG1 (i.v., 20 mg/kg) was started when the tumor volume in mice reached 220–280 mm^3^. Tumor growth and weight was performed. Tumors were dissected at the end of the treatment and used for further processing for RNA, protein, and histological analyses.

**Preparation of patient-derived tumor cells**

Tumor tissues from PDXs were washed with basic RPMI1640 medium (Gibco, USA. Free-FBS medium). Patient-derived tumor cells (PDC) were prepared from cryopreserved xenograft fragments using a Tumor Dissociation Kit (Miltenyi Biotec, Germany) following the protocol for tumors. PDCs culture was performed as described by Bruna and Shanker *et. al.*[^15^](#_ENREF_15)^,^ [^16^](#_ENREF_16). PDCs were used for Colony formation assay.

**RNA Interference**

The small interfering RNAs (siRNAs) against both human *ZIC2* and mouse *Zic2* were synthesized by Rio&Bio Biotech (Guangzhou, China). For siRNAs transfection, the cells were transfected with the indicated siRNAs using Lipofectamine 2000 (Invitrogen, USA), following the manufacturer’s instructions. si-*ZIC2* sequences were described in **Supplementary Table 9**.

**Chromatin immunoprecipitation (ChIP) assay**

A375, B16-F10 melanoma cells and HEK293T cells were grown to 80% confluency. ChIP was performed using the Simple ChIP Enzymatic Chromatin IP kit (Cell Signaling Technology, USA) according to the manufacturer’s directions. Native chromatin immunoprecipitation was performed overnight with the target antibody. The eluted DNAs were amplified by real-time PCR (detailed primer pairs information in **Supplementary Table 9**).

**RNA immunoprecipitation (RIP) assays**

We performed RIP assays using the Magna RIP RNA-Binding Protein Immunoprecipitation Kit (Millipore, Bedford, MA, USA) according to the manufacturer’s instructions. The G4 specific antibody BG4 (absolute antibody, UK) was used for RIP assay. The coprecipitated RNAs were detected by qRT-PCR assays. Total RNAs (input controls) and isotype controls were assayed simultaneously to confirm that the detected signals were from RNAs specifically binding to BG4. Data represent the mean ± SEM of three independent experiments.

**Statistical analysis**

All statistical analyses were performed using GraphPad Prism software. Survival curves were calculated using Kaplan–Meier and log-rank tests. Student’s *t*-test and multi-way classification ANOVA tests were performed for results from Luciferase reporter, qRT-PCR, WB (western blot), CCK-8, clolony formation, scratch-wound, cell invasion, ChIP-qPCR, immunofluorescence, tumor growth and metastasis assay. *P* < 0.05 was considered to be statistically significant. All data reported in this work are from at least three independent experiments.

**Supplementary References**

1. Shen, Y.A. *et al.* Development of small molecule inhibitors targeting PBX1 transcription signaling as a novel cancer therapeutic strategy. *iScience* **24**, 103297 (2021).

2. Shiraishi, K. *et al.* Pre-B-cell leukemia transcription factor 1 is a major target of promyelocytic leukemia zinc-finger-mediated melanoma cell growth suppression. *Oncogene* **26**, 339-348 (2007).

3. Liu, Y. *et al.* Ablation of H(+)/glucose Exporter SLC45A2 Enhances Melanosomal Glycolysis to Inhibit Melanin Biosynthesis and Promote Melanoma Metastasis. *J. Invest. Dermatol.* **10**, 2744-2755.e9 (2022).

4. Patel, B.R. & Tall, G.G. Ric-8A gene deletion or phorbol ester suppresses tumorigenesis in a mouse model of GNAQ(Q209L)-driven melanoma. *Oncogenesis* **5**, e236 (2016).

5. Phan, H.T.N., Kim, N.H., Wei, W., Tall, G.G. & Smrcka, A.V. Uveal melanoma-associated mutations in PLCbeta4 are constitutively activating and promote melanocyte proliferation and tumorigenesis. *Sci. Signal.* **14**, eabj4243 (2021).

6. Kikin, O., D'Antonio, L. & Bagga, P.S. QGRS Mapper: a web-based server for predicting G-quadruplexes in nucleotide sequences. *Nucleic Acids Res.* **34**, W676-682 (2006).

7. Labudova, D., Hon, J. & Lexa, M. pqsfinder web: G-quadruplex prediction using optimized pqsfinder algorithm. *Bioinformatics* **36**, 2584-2586 (2020).

8. Garant, J.M., Perreault, J.P. & Scott, M.S. G4RNA screener web server: User focused interface for RNA G-quadruplex prediction. *Biochimie* **151**, 115-118 (2018).

9. Beaudoin, J.D., Jodoin, R. & Perreault, J.P. New scoring system to identify RNA G-quadruplex folding. *Nucleic Acids Res.* **42**, 1209-1223 (2014).

10. Bedrat, A., Lacroix, L. & Mergny, J.L. Re-evaluation of G-quadruplex propensity with G4Hunter. *Nucleic Acids Res.* **44**, 1746-1759 (2016).

11. Garant, J.M., Perreault, J.P. & Scott, M.S. Motif independent identification of potential RNA G-quadruplexes by G4RNA screener. *Bioinformatics* **33**, 3532-3537 (2017).

12. Messeguer, X. *et al.* PROMO: detection of known transcription regulatory elements using species-tailored searches. *Bioinformatics* **18**, 333-334 (2002).

13. Dewaele, M. *et al.* Antisense oligonucleotide-mediated MDM4 exon 6 skipping impairs tumor growth. *J. Clin. Invest.* **126**, 68-84 (2016).

14. Han, Y. *et al.* Repurposing Ponatinib as a Potent Agent against KIT Mutant Melanomas. *Theranostics* **9**, 1952-1964 (2019).

15. Bruna, A. *et al.* A Biobank of Breast Cancer Explants with Preserved Intra-tumor Heterogeneity to Screen Anticancer Compounds. *Cell* **167**, 260-274 e222 (2016).

16. Hansel-Hertsch, R. *et al.* Landscape of G-quadruplex DNA structural regions in breast cancer. *Nat. genet.* **52**, 878-883 (2020).

**Supplementary tables and figures**

**Supplementary Table S1.** Major demographic and clinicopathological parameters of 47 pairs melanoma patients.

**Supplementary Table S2.** Major demographic and clinicopathological parameters of 48 melanoma patients and 15 normal tissues.

**Supplementary Table S3.** Differentially expressed genes upon PBX1 overexpression.

**Supplementary Table S4.** Characteristics of the 5 putative G-quadruplexes.

**Supplementary Table S5.** PBX1 G-quadruplexes and their G/A mutant sequences.

**Supplementary Table S6.** CD Melting temperatures of dG1 and rG1 oligonucleotides.

**Supplementary Table S7.** Antisense oligonucleotide sequences in this study.

**Supplementary Table S8.** PDX xenopatient information.

**Supplementary Table S9.** Primers, shRNAs and siRNAs in this study.

**Supplementary Figure S1.** PBX1 pathological parameters and functions in melanoma.

**Supplementary Figure S2.** Transcriptome-sequencing identified the downstream signaling pathways of PBX1 in melanoma.

**Supplementary Figure S3.** Effects of PBX1 on NF-κB signaling pathway in melanoma.

**Supplementary Figure S4.** Identification of G4s in genomic PBX1 locus and PBX1 transcripts.

**Supplementary Figure S5.** Characterization of dG1 and rG1 formation *in vitro* and in cells.

**Supplementary Figure S6.** PDS and TMPyP4 treatment inhibit PBX1 expression in B16-F10 cells.

**Supplementary Figure S7.** PDS and TMPyP4 suppress melanoma progression *in vitro*.

**Supplementary Figure S8.** PDS suppress melanoma progression *in* *vivo*.

**Supplementary Figure S9.** PDS and TMPyP4 suppress B16-F10 cells-derived xenograft tumor growth *in* *vivo*.

**Supplementary Figure S10.** Prediction of the RNA secondary structure of PBX1 5’UTR.

**Supplementary Figure S11.** PBX1 rG1 specific ASO inhibits melanoma progression.

**Supplementary Figure S12.** Knockout of *PBX1* blocks the PBX1 rG1 specific ASO anti-melanoma effects.

**Supplementary Figure S13.** PBX1-overexpressed melanoma cells were more sensitive to G4 ligands (PDS and TMPyP4) and ASO.

**Supplementary Figure S14.** Mechanisms of PBX1 G4s on regulating expression of PBX1.

**Supplementary Tables**

| **Supplementary Table 1. Major demographic and clinicopathological parameters of 47 melanoma patients.** | | | | | | |  |
| --- | --- | --- | --- | --- | --- | --- | --- |
| **Samples** | **Ages** | **Sexs** | **Ucleration（yes/no）** | **Breslow thickness(＜0.5cm/0.5-1.0cm/＞1.0cm)** | **Clark level** | **Tumor-Infiltrating Lymphocytes(no/yes/brisk)** |  |
|  |  |  |  |  |  |  |  |
| No.1 | 64 | Male | No | ＞1.0 mm | 4 | No |  |
| No.2 | 79 | Male | No | ＞1.0 mm | 4 | No |  |
| No.3 | 65 | Male | No | ＞1.0 mm | 4 | No |  |
| No.4 | 79 | Female | Yes | ＞1.0 mm | 4 | Yes |  |
| No.5 | 81 | Female | Yes | ＞1.0 mm | 4 | Yes |  |
| No.6 | 54 | Female | Yes | ＞1.0 mm | 4 | Yes |  |
| No.7 | 67 | Female | Yes | ＞1.0 mm | 3 | Brisk |  |
| No.8 | 71 | Female | No | ＞1.0 mm | 2 | Yes |  |
| No.9 | 83 | Male | No | ＞1.0 mm | 4 | No |  |
| No.10 | 36 | Male | No | 0.5-1.0 mm | 4 | Yes |  |
| No.11 | 67 | Male | No | ＞1.0 mm | 3 | Yes |  |
| No.12 | 80 | Male | No | ＞1.0 mm | 4 | Yes |  |
| No.13 | 73 | Male | No | ＞1.0 mm | 3 | Brisk |  |
| No.14 | 79 | Male | Yes | ＞1.0 mm | 4 | Yes |  |
| No.15 | 80 | Male | Yes | ＞1.0 mm | 4 | Yes |  |
| No.16 | 65 | Male | No | ＞1.0 mm | 4 | Yes |  |
| No.17 | 46 | Male | Yes | ＞1.0 mm | 3 | No |  |
| No.18 | 94 | Male | Yes | ＞1.0 mm | 3 | Yes |  |
| No.19 | 65 | Female | Yes | ＞1.0 mm | 5 | Yes |  |
| No.20 | 87 | Female | No | ＞1.0 mm | 4 | No |  |
| No.21 | 66 | Female | No | ＞1.0 mm | 4 | Yes |  |
| No.22 | 51 | Male | Yes | ＞1.0 mm | 4 | Yes |  |
| No.23 | 65 | Male | No | ＞1.0 mm | 4 | Yes |  |
| No.24 | 53 | Female | No | ＞1.0 mm | 4 | Yes |  |
| No.25 | 67 | Male | No | ＞1.0 mm | 4 | No |  |
| No.26 | 58 | Female | Yes | ＞1.0 mm | 3 | Brisk |  |
| No.27 | 79 | Female | Yes | ＞1.0 mm | 4 | Yes |  |
| No.28 | 61 | Female | No | regression | regression | Yes |  |
| No.29 | 53 | Female | No | ＜0.5 mm | 2 | No |  |
| No.30 | 88 | Male | No | ＞1.0 mm | 2 | Yes |  |
| No.31 | 73 | Male | No | ＜0.5 mm | 4 | No |  |
| No.32 | 72 | Male | No | ＞1.0 mm | 4 | No |  |
| No.33 | 38 | Female | No | ＜0.5 mm | 3 | No |  |
| No.34 | 78 | Male | Yes | ＜0.5 mm | 4 | No |  |
| No.35 | 50 | Male | Yes | ＜0.5 mm | 4 | Yes |  |
| No.36 | 56 | Male | Yes | ＜0.5 mm | 4 | No |  |
| No.37 | 50 | Male | Yes | ＜0.5 mm | 4 | No |  |
| No.38 | 65 | Female | No | 0.5-1.0 mm | 4 | No |  |
| No.39 | 84 | Female | No | 0.5-1.0 mm | 4 | No |  |
| No.40 | 67 | Male | No | 0.5-1.0 mm | 4 | No |  |
| No.41 | 87 | Female | No | ＜0.5 mm | 3 | No |  |
| No.42 | 66 | Male | No | 0.5-1.0 mm | 2 | No |  |
| No.43 | 58 | Male | Yes | 0.5-1.0 mm | 4 | No |  |
| No.44 | 64 | Male | Yes | ＜0.5 mm | 3 | No |  |
| No.45 | 63 | Male | Yes | ＜0.5 mm | 4 | No |  |
| No.46 | 49 | Male | Yes | ＜0.5 mm | 3 | No |  |
| No.47 | 80 | Female | Yes | ＜0.5 mm | 4 | No |  |

| **Supplementary Table 2. Major demographic and clinicopathological parameters of 48 melanoma patients and 15 normal tissues.** | | | | | | |  |
| --- | --- | --- | --- | --- | --- | --- | --- |
| **Samples** | **Ages** | **Sexs** | **Pathological diagnosis** | **TNM level** | **Survival status** | **Survival time (months)** |  |
|  |  |  |  |  |  |  |  |
| No.1 | 45 | Female | Malignant melanoma (left chest wall) | T3bN0M0 | Alive | 37 |  |
| No.2 | 62 | Female | Malignant melanoma (right foot) | T4aN0M0 | Alive | 62 |  |
| No.3 | 45 | Male | Malignant melanoma (perianal) | T3bN0M0 | Alive | 55 |  |
| No.4 | 80 | Male | Malignant melanoma (right plantar) | T4aN0M0 | Alive | 68 |  |
| No.5 | 46 | Female | Malignant melanoma (thigh) | T4bN0M0 | Alive | 23 |  |
| No.6 | 42 | Male | Malignant melanoma (left heel) | T3aN0M0 | Alive | 56 |  |
| No.7 | 74 | Female | Malignant melanoma of the back | T4aN0M0 | Alive | 30 |  |
| No.8 | 51 | Male | Malignant melanoma of the back | T4aN0M0 | Alive | 29 |  |
| No.9 | 37 | Male | Malignant melanoma (right upper arm) | T4aN0M0 | Alive | 63 |  |
| No.10 | 61 | Male | Malignant melanoma (right groin) | T4aN3M1a | Alive | 16 |  |
| No.11 | 47 | Female | Malignant melanoma (right lower leg) | T4aN0M0 | Alive | 19 |  |
| No.12 | 66 | Female | Malignant melanoma of the abdominal wall | T4bN0M0 | Alive | 25 |  |
| No.13 | 45 | Female | Malignant melanoma (left plantar) | T4aN0M0 | Alive | 28 |  |
| No.14 | 35 | Female | Malignant melanoma of the lower back | T4aN3M1a | Alive | 72 |  |
| No.15 | 47 | Female | Malignant melanoma (right dorsolis) | T4aN0M0 | Alive | 75 |  |
| No.16 | 76 | Male | Malignant melanoma (right anterior tibial) | T4aN0M0 | Dead | 10 |  |
| No.17 | 72 | Female | Malignant melanoma tissue (left thigh root) | T4aN0M0 | Dead | 18 |  |
| No.18 | 74 | Female | Malignant melanoma (left lower limb) | T4aN3M1a | Alive | 32 |  |
| No.19 | 63 | Male | Malignant melanoma (left occipital scalp) | T4aN0M0 | Alive | 47 |  |
| No.20 | 61 | Male | Malignant melanoma with necrosis (deep right lateral malleolus) | T4aN0M0 | Alive | 21 |  |
| No.21 | 71 | Female | Malignant melanoma (medial left thigh) | T4aN0M0 | Alive | 25 |  |
| No.22 | 62 | Female | Malignant melanoma (left plantar) | T4aN0M0 | Dead | 7 |  |
| No.23 | 61 | Male | Malignant melanoma (left plantar) | T4aN0M0 | Dead | 15 |  |
| No.24 | 83 | Female | Malignant melanoma with necrosis (left foot thumb) | T4aN0M0 | Dead | 17 |  |
| No.25 | 71 | Male | Malignant melanoma (right hip) | T4aN0M0 | Dead | 14 |  |
| No.26 | 38 | Female | Malignant melanoma (right shoulder) | T4aN1M0 | Dead | 16 |  |
| No.27 | 49 | Male | Malignant melanoma (left thigh) | T4aN0M0 | Dead | 12 |  |
| No.28 | 42 | Female | Malignant melanoma (right thigh) | T4aN0M0 | Dead | 19 |  |
| No.29 | 7 | Male | Malignant melanoma (sacral caudal region) | T4aN0M0 | Alive | 33 |  |
| No.30 | 70 | Female | Malignant melanoma (left parotid gland) | - | Alive | 47 |  |
| No.31 | 50 | Male | Malignant melanoma (esophagus) | - | Dead | 80 |  |
| No.32 | 62 | Female | Malignant melanoma (urinary tract) | - | Dead | 77 |  |
| No.33 | 47 | Female | Malignant melanoma (nasal cavity) | - | Dead | 75 |  |
| No.34 | 63 | Male | Malignant melanoma (left maxillary sinus) | - | Alive | 23 |  |
| No.35 | 48 | Female | Malignant melanoma (mediastinum) | - | Alive | 26 |  |
| No.36 | 46 | Male | Malignant melanoma (scrotum) | T4aN0M0 | Dead | 33 |  |
| No.37 | 62 | Male | Malignant melanoma (scrotum) | T4bN0M0 | Dead | 16 |  |
| No.38 | 37 | Male | Malignant melanoma (choroid) | T3N1M0 | Dead | 10 |  |
| No.39 | 46 | Female | Metastatic malignant melanoma (groin) | - | Dead | 25 |  |
| No.40 | 45 | Male | Metastatic malignant melanoma (right axilla) | - | Dead | 43 |  |
| No.41 | 40 | Female | Metastatic malignant melanoma (palate) | - | Alive | 61 |  |
| No.42 | 61 | Female | Metastatic malignant melanoma (right groin) | - | Alive | 78 |  |
| No.43 | 65 | Male | Metastatic malignant melanoma (groin) | - | Alive | 75 |  |
| No.44 | 48 | Female | Metastatic malignant melanoma (mediastinum) | - | Alive | 77 |  |
| No.45 | 73 | Male | Metastatic malignant melanoma (small intestine) | - | Alive | 52 |  |
| No.46 | 70 | Male | Metastatic malignant melanoma (armpit) | - | Alive | 60 |  |
| No.47 | 51 | Female | Metastatic malignant melanoma (right lobe of liver) | - | Alive | 32 |  |
| No.48 | 49 | Male | Metastatic malignant melanoma (left axilla) | - | Dead | 26 |  |
| No.49 | 56 | Female | Skin tissue (normal) | - | - | - |  |
| No.50 | 35 | Male | Skin tissue (normal) | - | - | - |  |
| No.51 | 28 | Male | Normal skin tissue (scalp) | - | - | - |  |
| No.52 | 40 | Female | Normal skin tissue (scalp) | - | - | - |  |
| No.53 | 42 | Female | Normal skin tissue (scalp) | - | - | - |  |
| No.54 | 30 | Male | Normal skin tissue (scalp) | - | - | - |  |
| No.55 | 40 | Male | Normal skin tissue (abdomen) | - | - | - |  |
| No.56 | 36 | Male | Mucosa of pharynx | - | - | - |  |
| No.57 | 48 | Male | Mucosa of pharynx | - | - | - |  |
| No.58 | 28 | Male | Esophageal mucosa tissue | - | - | - |  |
| No.59 | 40 | Female | Esophageal mucosa tissue | - | - | - |  |
| No.60 | 45 | Male | Small intestinal mucosa | - | - | - |  |
| No.61 | 40 | Male | Small intestinal mucosa | - | - | - |  |
| No.62 | 30 | Male | Lymph node tissue (axillary) | - | - | - |  |
| No.63 | 33 | Female | Lymph node tissue (neck) | - | - | - |  |
| Note：-, in "TNM level, Survival status and Survival time (months)", it indicates not applicable. | | | | | | |  |

| **Supplementary Table 4. Characteristics of the 5 putative G-quadruplexes.** | | |
| --- | --- | --- |
| **Putative G-quadruplexes** | **Positions** | **Sequences** |
| dG1 | Promoter (+146; +176) | TGGGGGTGGGGGGCAGCGGGGGGTGGGGGGGGA |
| dG2 | Promoter (-134; -115) | AGGGAAGGGGAGGGGGCGGGGA |
| rG1 | mRNA-5'UTR (146; 176) | UGGGGGUGGGGGGCAGCGGGGGGUGGGGGGGGA |
| rG2 | mRNA-CDS (733; 753) | UGGGGCCGGAGGGACCGAGGGGGAGGGCGGGA |
| rG3 | mRNA-3'UTR (1,975; 1,995) | UGGGGCAGGGGCAGGAGGGAGGGU |
| CDS, coding sequences; UTR, untranslated region. | | |

| **Supplementary Table 5. PBX1 G-quadruplexes and their G/A mutant sequences.** | |
| --- | --- |
| **Putative G-quadruplexes** | **Sequences** |
| dG1-WT | TGGGGGTGGGGGGCAGCGGGGGGTGGGGGGGGA |
| dG1-Mut | TGGAAGTGAAAGGCAGCGAAAGGTGAAAGGGGA |
| rG1-WT | UGGGGGUGGGGGGCAGCGGGGGGUGGGGGGGGA |
| rG1-Mut | UGGAAGUGAAAGGCAGCGAAAGGUGAAAGGGGA |

| **Supplementary Table 6. CD Melting temperatures of dG1 and rG1 oligonucleotides** | | | | |
| --- | --- | --- | --- | --- |
| **PBX1 GQs** | **K^+^** | **GQs ligands** | ***T*_m_^a^** | **Δ *T*_m_^a^** |
| dG1 | 10 mM | **-** | 73.5 ± 1.6 ℃ | **-** |
| dG1 | 10 mM | TMPyP4 | 78.9 ± 0.8 ℃ | 5.4 ± 2.8 ℃ |
| dG1 | 10 mM | PDS | 81.2 ± 0.6 ℃ | 7.7 ± 2.2 ℃ |
| rG1 | 10 mM | - | 85.7 ± 2.1 ℃ | - |
| rG1 | 10 mM | TMPyP4 | > 90 ℃ | > 4.3 ℃ |
| rG1 | 10 mM | PDS | > 90 ℃ | > 4.3 ℃ |
| ^a^The reported *T*_m_ values were calculated at the positive peak (∼261–265 nm) and represent the mean of three independent replicates. GQs, G-quadruplexes | | | |  |

| **Supplementary Table 7. Antisense oligonucleotide sequences in this study.** | | |
| --- | --- | --- |
| **Sequences of antisense oligonucleotide** | | |
| ASO ID | Length | Sequence |
| ASO Scr | 20 | mG∗mT∗mC∗mC∗mA∗mC∗mA∗mA∗mA∗mC∗mA∗mC∗mA∗mA∗mC∗mT∗mC∗mC∗mT∗mG |
| ASO PBX1 | 20 | mG∗mG∗mG∗mG∗mT∗mT∗mG∗mC∗mG∗mG∗mG∗mG∗mT∗mG∗mA∗mG∗mG∗mG∗mT∗mG |
| m represents 2`-O-Methyl RNA modification; ∗ represents phosphorothioate linkage modification; ASO Scr represents ASO Scramble | | |

| **Supplementary Table 8. PDX xenopatient information.** | | | | | | | | |  |
| --- | --- | --- | --- | --- | --- | --- | --- | --- | --- |
| **Samples** | **Sexs** | **Ucleration（yes/no）** | **Breslow thickness(＜0.5mm/0.5-1.0mm/＞1.0mm)** | **Melanoma Stage** | **Tumor-Infiltrating Lymphocytes(no/yes/brisk)** | **Pathological diagnosis** | **BRAF V600 status** | **Treatment prior（yes/no）** |  |
|  |  |  |  |  |  |  |  |  |  |
| No.1 | Male | Yes | ＜0.5mm | Melanoma in situ | No | Malignant melanoma (left temporal lobe) | V600E | No |  |
| No.2 | Male | Yes | ＜0.5mm | Melanoma in situ | No | Malignant melanoma (right foot) | V600E | No |  |
| No.3 | Male | Yes | 0.5-  1.0mm | Melanoma in situ | No | Malignant melanoma (left heel) | V600E | No |  |
| No.4 | Male | No | ＜0.5mm | Melanoma in situ | No | Malignant melanoma (left plantar) | V600E | No |  |
| No.5 | Male | No | ＜0.5mm | Melanoma in situ | No | Malignant melanoma (left foot) | V600E | No |  |
| No.6 | Male | Yes | ＜0.5mm | Melanoma in situ | No | Malignant melanoma (left plantar) | V600E | No |  |
| No.7 | Male | Yes | ＜0.5mm | Melanoma in situ | No | Malignant melanoma (left temporal lobe) | V600E | No |  |

| **Supplementary Table 9. Primers, shRNAs and siRNAs in this study.** | | |
| --- | --- | --- |
| **qRT-PCR primers for quantification** | | |
| *PBX1*_F | AGTTTGCAGAGACACGGAGG | *PBX1* mRNA quantification (Human) |
| *PBX1*_R | GTGATCTCAAAGGGGGTGGG |  |
| *Pbx1*_F | CTTCTTCTACCTGACCCGTGG | *Pbx1* mRNA quantification (Mouse) |
| *Pbx1*_R | CATCTGCTAGCTCCCTGCTC |  |
| *ZIC2*_F | AAAAGGACCCACACAGGGGA | *ZIC2* mRNA quantification (Human) |
| *ZIC2*_R | GAGGATTCAGAGCCCTGCG |  |
| *Zic2*_F | AACAGCAGCGACAGGAAGAA | *Zic2* mRNA quantification (Mouse) |
| *Zic2*_R | CTGAGGGGAGGACTCATGGA |  |
| *CXCL2*_F | AGATCAATGTGACGGCAGGG | *CXCL2* mRNA quantification (Human) |
| *CXCL2*_R | TCTCTGCTCTAACACAGAGGGA |  |
| *IL6*_F | TACAGGGAGAGGGAGCGATAA | *IL6* mRNA quantification (Human) |
| *IL6*_R | GGGCGGCTACATCTTTGGAA |  |
| *ACKR3*_F | ATTTGATTGCCCGCCTCAGA | *ACKR3* mRNA quantification (Human) |
| *ACKR3*_R | GACGCTTTTGTTGGGCATGT |  |
| *BIRC3*_F | GCATTTAAAAGACAGCGTGAGACT | *BIRC3* mRNA quantification (Human) |
| *BIRC3*_R | TCAGGCCACAACAGAAGCAT |  |
| *PTX3*_F | CCTGCATTTGGGTCAAAGCC | *PTX3* mRNA quantification (Human) |
| *PTX3*_R | AGCCGCCAGTTCACCATTTA |  |
| *GAPDH*_F | CGGAGTCAACGGATTTGGTCGT | *GAPDH* mRNA quantification (Human) |
| *GAPDH*_R | TCTCAGCCTTGACGGTGCCA |  |
| *Gapdh*_F | CAGGTTGTCTCCTGCGACTT | *Gapdh* mRNA quantification (Mouse) |
| *Gapdh*_R | TATGGGGGTCTGGGATGGAA |  |
| **ChIP-qRT-PCR primers** | | |
| *EIF4A* ChIP_F | CCGGAGCGACTAGGAACTAAC | ChIP-qRT-PCR primers for *EIF4A* |
| *EIF4A* ChIP_R | GCCTTTCTTACCGGGAATCCT |  |
| *TMCC2* ChIP_F | CCAGACACTTTGGGTGACCT | ChIP-qRT-PCR primers for *TMCC2* |
| *TMCC2* ChIP_R | AACACCTGCTCTGCCAACTT |  |
| *PBX1* ChIP_F | TCAGTTCACTTCTCTTCCCAGTT | ChIP-qRT-PCR primers for *PBX1* (Human) |
| *PBX1* ChIP_R | GCACAAGTTTGTCAGCACGA |  |
| *Pbx1* ChIP_F | TGGCATAGAGTTGAGGATTTGC | ChIP-qRT-PCR primers for *Pbx1* (Mouse) |
| *Pbx1* ChIP_R | CTCCGTCCTTGAGGGAAACG |  |
| **RIP-qRT-PCR primers** | | |
| *NRAS* RIP_F | CCTCCTCACTTGGCTGTCTG | ChIP-qRT-PCR primers for *NRAS* |
| *NRAS* RIP_R | TCACGTTTGCGGTTTGGTTC |  |
| *PBX1* RIP_F | AGTTTGCAGAGACACGGAGG | ChIP-qRT-PCR primers for *PBX1* |
| *PBX1* RIP_R | GTGATCTCAAAGGGGGTGGG |  |
| *ACTB* RIP_F | AGAGCCTCGCCTTTGCCGAT | ChIP-qRT-PCR primers for *ACTB* |
| *ACTB* RIP_R | CCATCACGCCCTGGTGCCT |  |
| **Sequences of shRNAs or siRNAs** | |  |
| *ZIC2* siRNA-1 | CCAACTTCAATGAATGGTA |  |
| *ZIC2* siRNA-2 | CTCCTCCAACTTCAATGAA |  |
| *ZIC2* siRNA-3 | GCCTCTCCTCCAACTTCAA |  |
| Scrambled control siRNA | Order number：PA20210411011 (RIO&BIO only offer the order number of scrambled control siRNA) | |
| ChIP, chromatin immunoprecipitation; F, forward primer; R, reverse primer; RIP, RNA immunoprecipitation; qRT-PCR, quantitative real-time polymerase chain reaction; RT-PCR, reverse transcription polymerase chain reaction. | | |

**
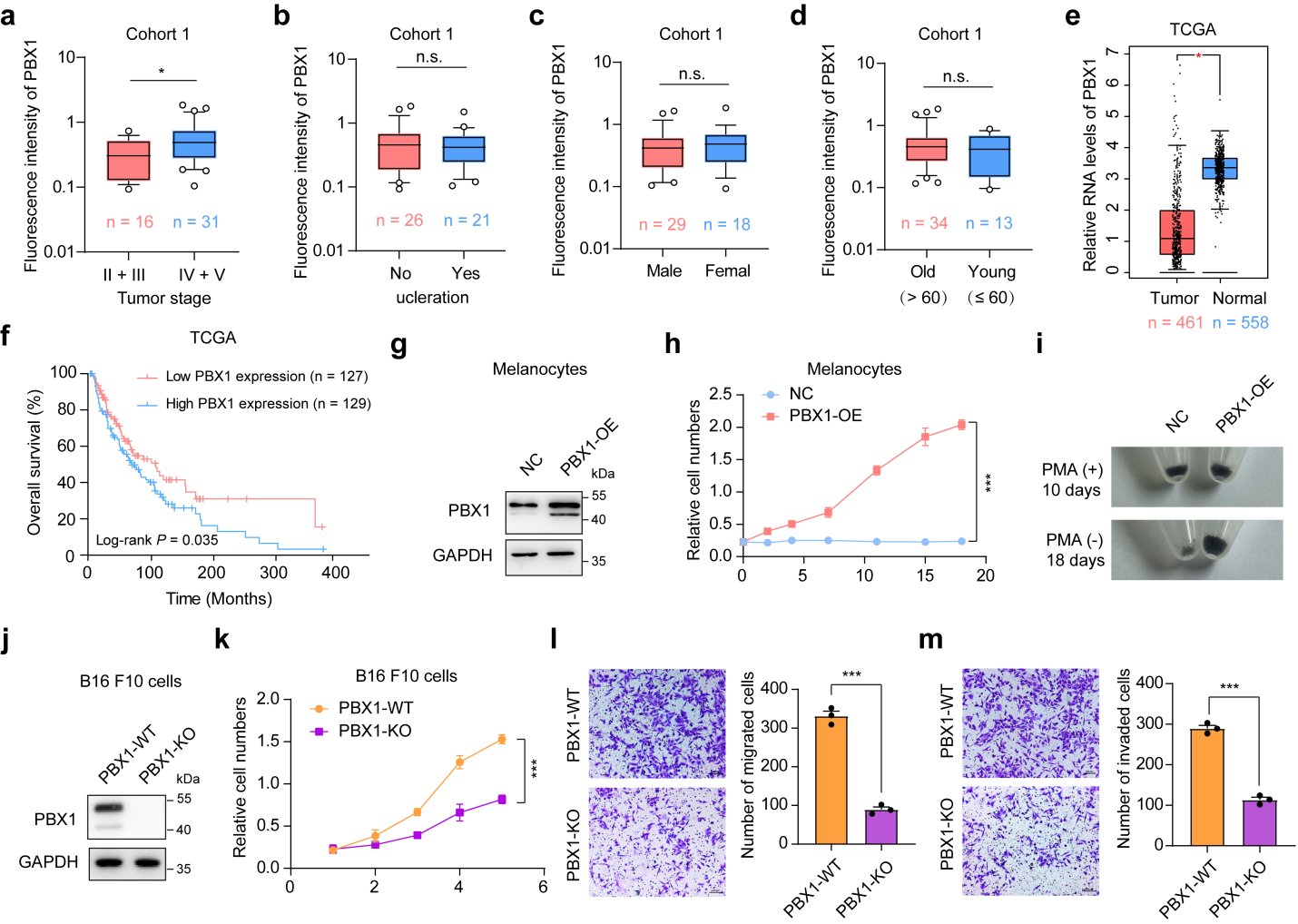
**

**Supplementary Fig. 1. PBX1 pathological parameters and functions in melanoma. a-d** The PBX1 expression of primary melanoma patient samples grouped by stage II + III versus IV + V samples (a), ulceration status (b), sex (c) and age (d). **e** The RNA levels of PBX1 in the melanoma and normal tissues from TCGA cohort. **f** The OS analyses for patients from the melanoma TCGA cohort. The high (blue) and low (red) expression of PBX1 in melanoma tissues were determined by the median of PBX1 expression levels in all melanoma samples. **g** The protein levels of PBX1 in melanocytes expressing pLVX-Ctrl (NC) or pLVX-PBX1 (PBX1-OE). **h** Cell proliferation of melanocytes expressing pLVX-Ctrl (NC) or pLVX-PBX1 (PBX1-OE). **i** Cell pellet photosof melanocytes expressing pLVX-Ctrl (NC) or pLVX-PBX1 (PBX1-OE), in melanocyte media supplemented with or without 200 nM phorbol 12-myristate 13-acetate (PMA) treatment for 10 or 18 days, respectively. **j** The protein levels of PBX1 in PBX1-wild type (WT) or PBX1-knockout (KO) B16-F10 cells. **k** Cell proliferation of PBX1 in PBX1-WT or PBX1-KO B16-F10 cells. **l,m C**ell migration (l) and invasion (m) of PBX1 in PBX1-WT or PBX1-KO B16-F10 cells. n.s., not significant. ^*^*P* < 0.05, ^***^*P* < 0.001.


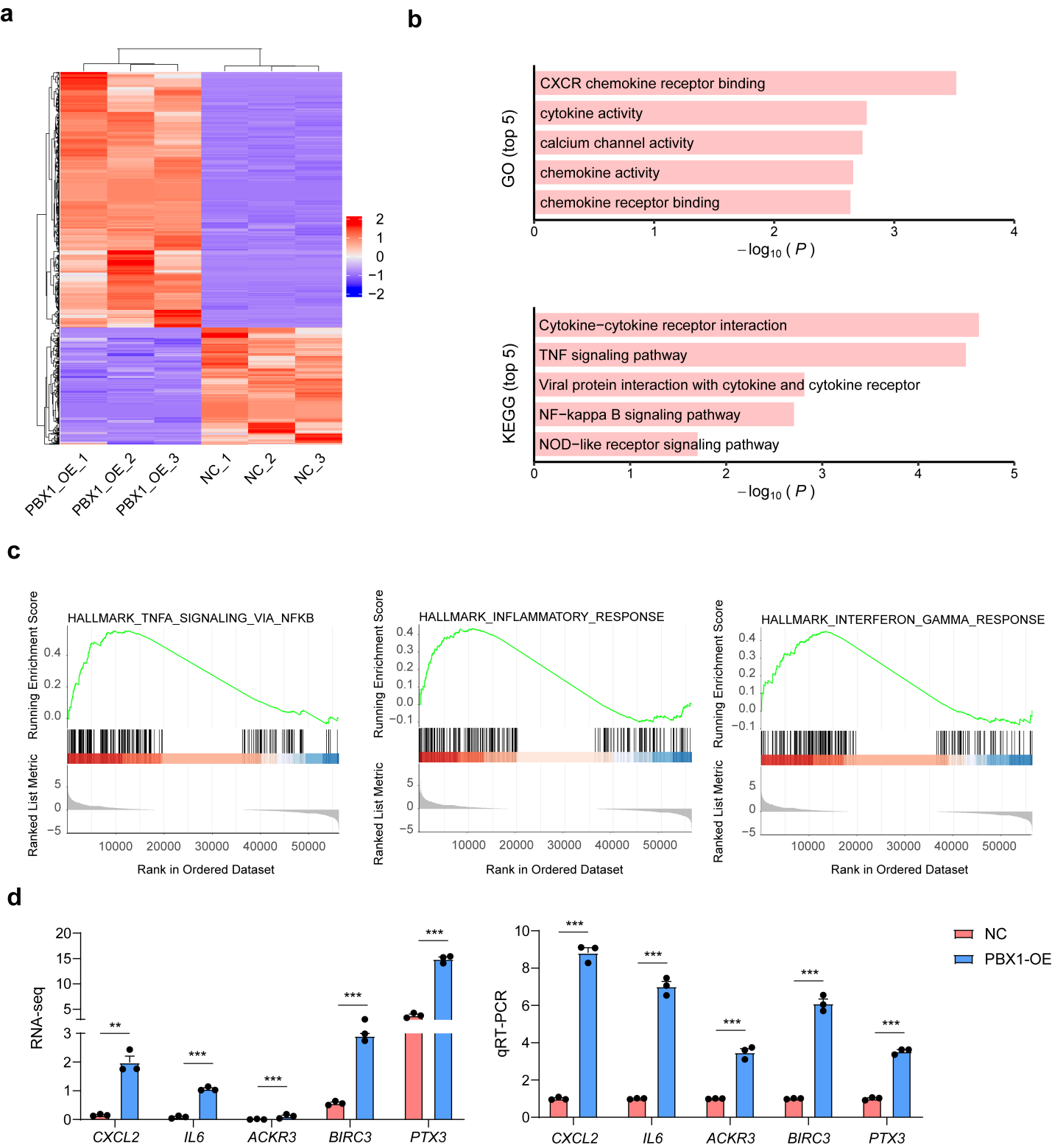


**Supplementary Fig. 2. Transcriptome-sequencing identified the downstream signaling pathways of PBX1 in melanoma. a** Expression heatmap of transcripts whose expressions were affected by PBX1 overexpression. Red and blue indicate up- and down-regulation, respectively. **b** Gene Ontology (GO) and Kyoto Encyclopedia of Genes and Genomes (KEGG) pathway enrichment analyses of changed genes in PBX1-overexpressed A375 cells. **c** Gene Set Enrichment Analysis (GSEA) of the PBX1-related pathways in A375 cells. **d** PBX1-regulated genes expression detected by RNA-seq (left) and qRT-PCR analyses (right) in A375 cells. Data are shown as mean ± SEM of three independent experiments, two-tailed Student’s t test. SEM, standard error of mean. ^**^*P* < 0.01, ^***^*P* < 0.001.

**
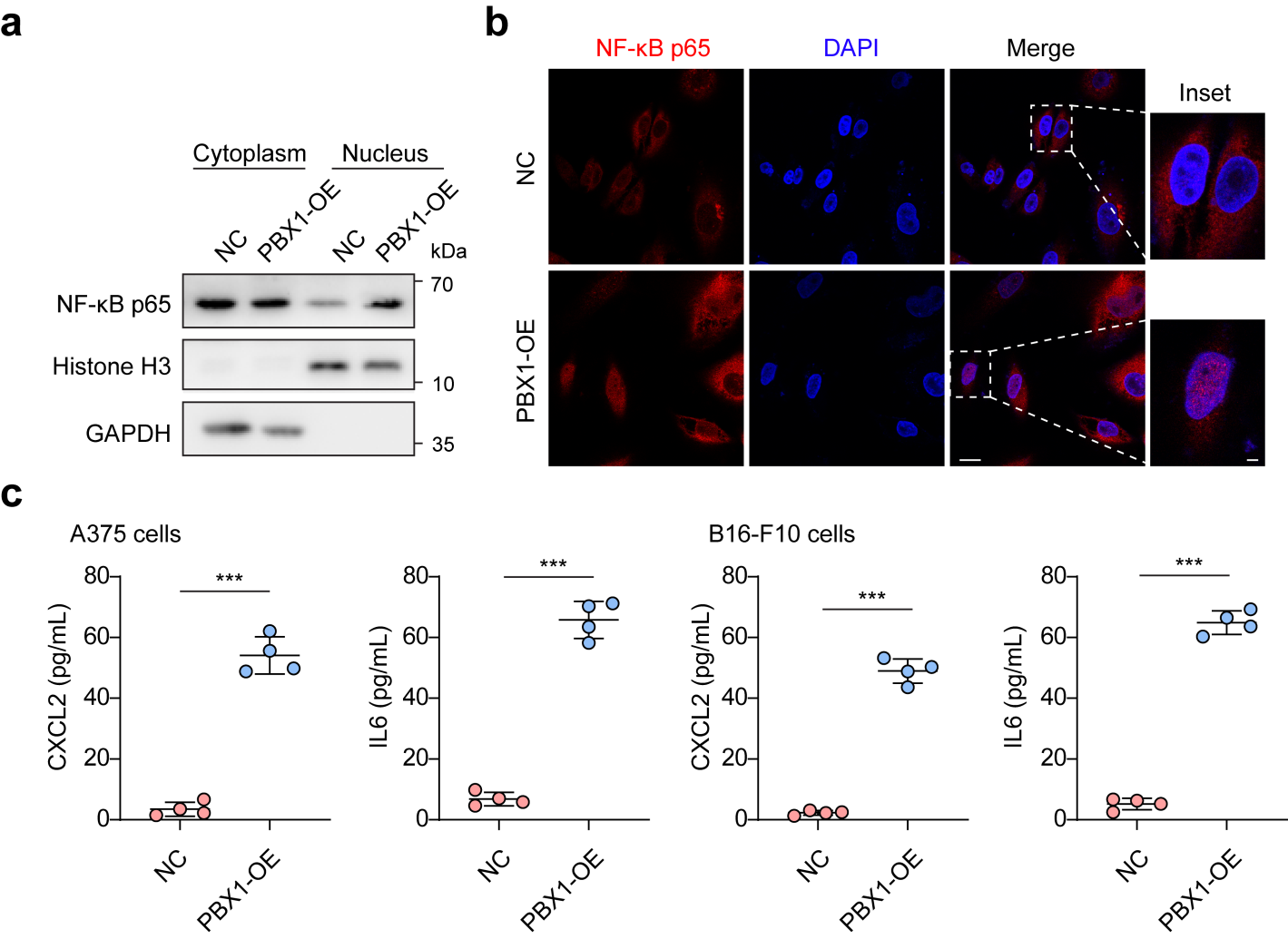
**

**Supplementary Fig. 3. Effects of PBX1 on NF-κB signaling pathway in melanoma. a** Nuclear translocation of NF-κB p65 upon PBX1 overexpression in A375 cells measured by western blot assays shows an increase in NF-κB translocation from cytoplasm to nucleus. **b** Nuclear translocation of NF-κB p65 upon PBX overexpression in A375 cells detected by immunofluorescence assays. Scale bars: 20 μm. The scale bars of inset: 5 μm. **c** The secreted CXCL2 and IL6 protein levels upon PBX1 overexpression measured by enzyme linked immunosorbent assay (ELISA). Data are shown as mean ± SEM, two-tailed Student’s t test. ^***^*P* < 0.001.

**
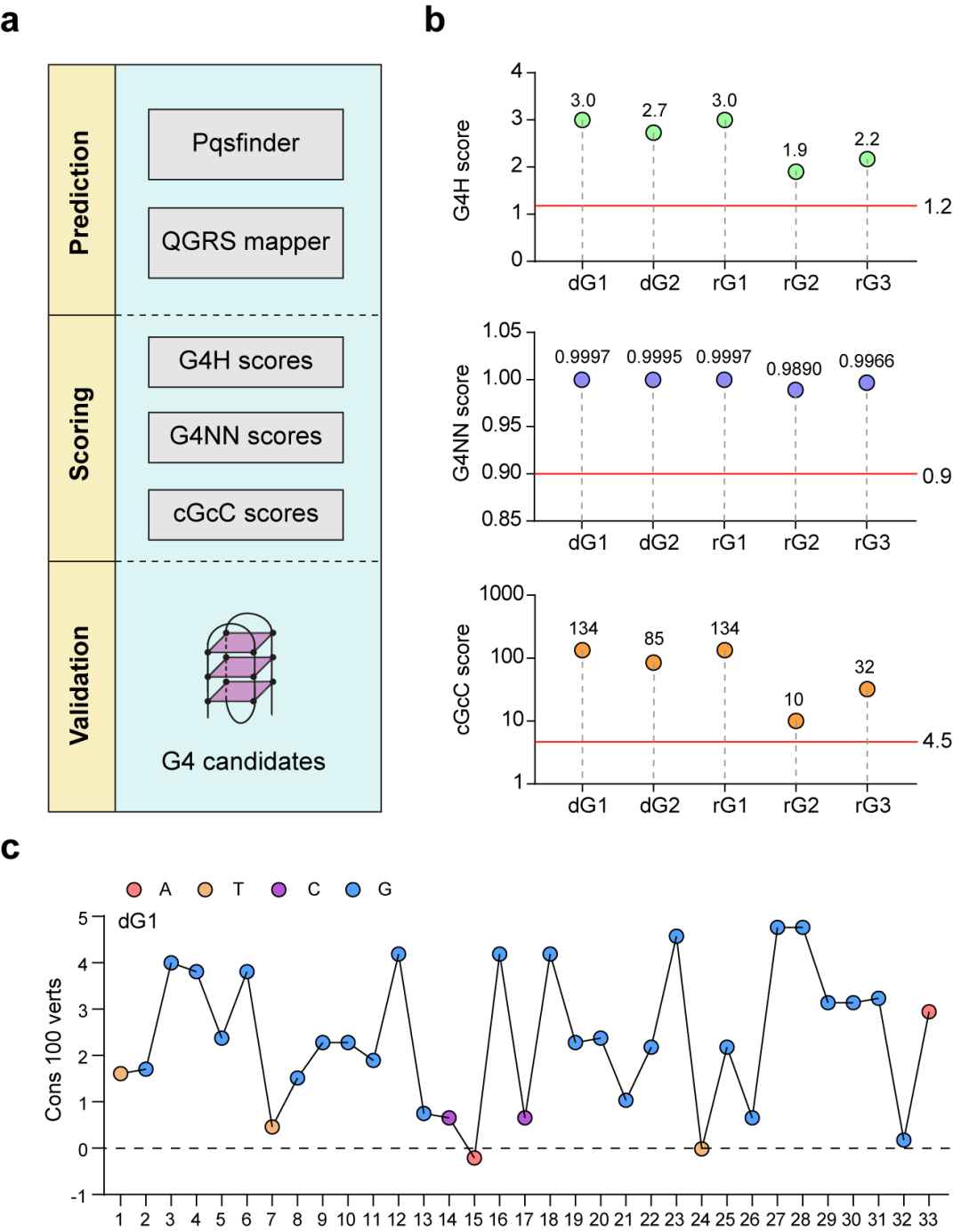
**

**Supplementary Fig. 4. Identification of G4s in genomic PBX1 locus and PBX1 transcripts. a** Schematic summary of the bioinformatic workflow conducted for PQSs identification in genomic PBX1 locus and PBX1 transcripts. **b** The G4H, G4NN and cGcC score of potential G4s in genomic PBX1 locus and PBX1 transcripts. The G4H, G4NN and cGcC score of G4s is calculated by using G4RNA screener. G4H, G4hunter; G4NN, G4 neural network; cGcC, cGcC, consecutive G over consecutive C ratio. **c** The analysis of conservation of the dG1 candidates throughout 100 species. Average PhastCons (100 Vert.) scores for dG1 sequence were obtained from the UCSC Genome Browser.

**
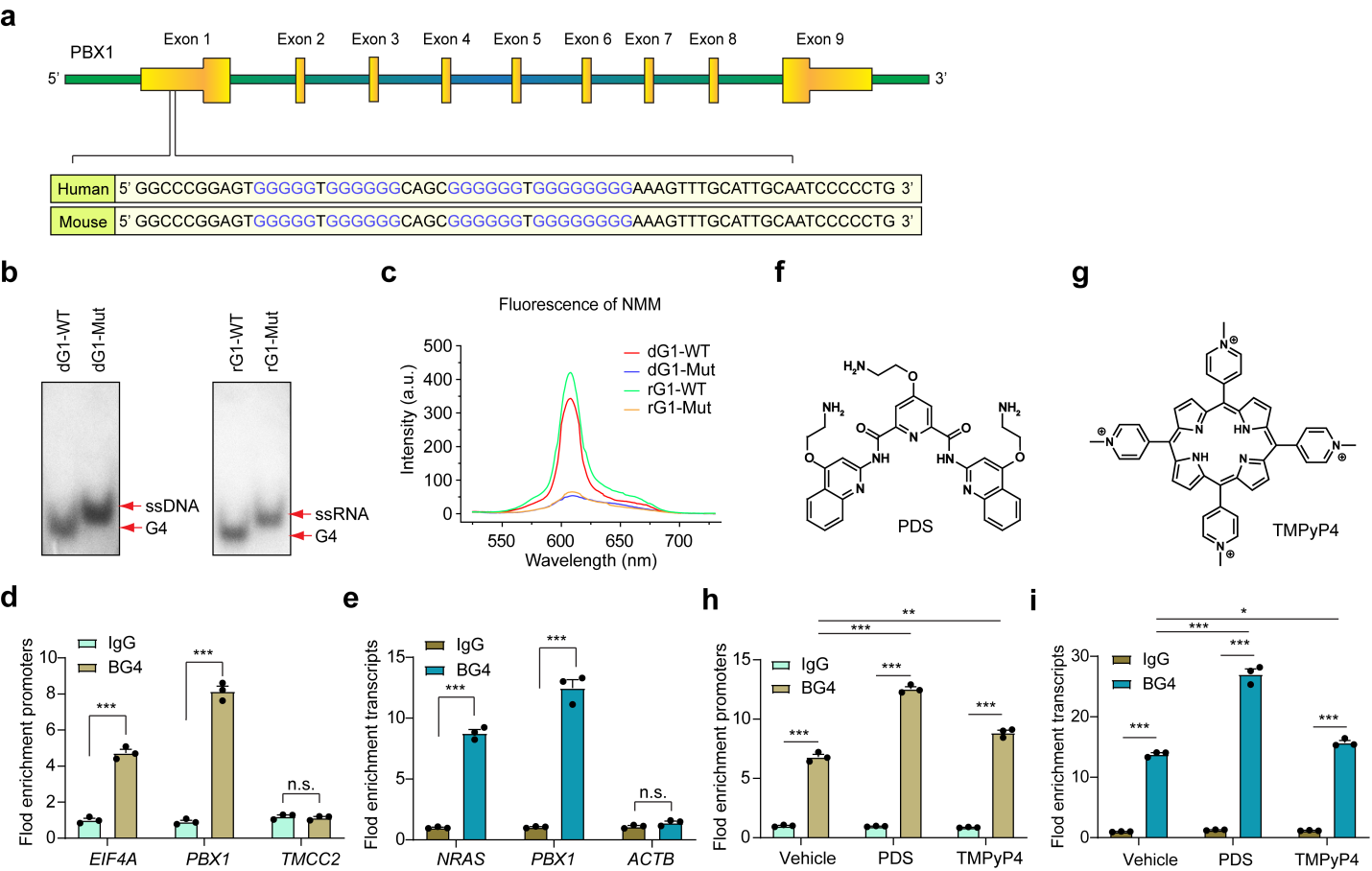
**

**Supplementary Fig. 5. Characterization of dG1 and rG1 formation *in vitro* and in cells. a** Location of G4s in the genomic site of *PBX1*. **b** Left: Gel mobility shift assay of WT and Mut dG1. Right: Gel mobility shift assay of WT and Mut rG1. **c** Fluorescence turn-on assays of NMM in the absence or presence of dG1 or rG1 and their G/A mutants under indicated conditions. **d** The occupancy of BG4 in the promoter region of PBX1 was measured by BG4 chromatin immunoprecipitation (ChIP) in A375 cells, followed by qRT-PCR. **e** RNA immunoprecipitation (RIP) assays show the association of BG4 with PBX1 transcripts in A375 cells, followed by qRT-PCR. **f,g** Chemical structures of PDS (f) and TMPyP4 (g). **h** The occupancy of BG4 in the promoter region of PBX1 was measured by BG4 ChIP in A375 cells with or without PDS (2 μM) and TMPyP4 (5 μM) treatment for 48 h, followed by qRT-PCR. **i** RIP assays show the association of BG4 with PBX1 transcripts in A375 cells with or without PDS (2 μM) and TMPyP4 (5 μM) treatment, followed by qRT-PCR. Data are shown as mean ± SEM of three independent experiments, two-tailed Student’s t test. SEM, standard error of mean. ^*^*P* < 0.05, ^**^*P* < 0.01, ^***^*P* < 0.001.


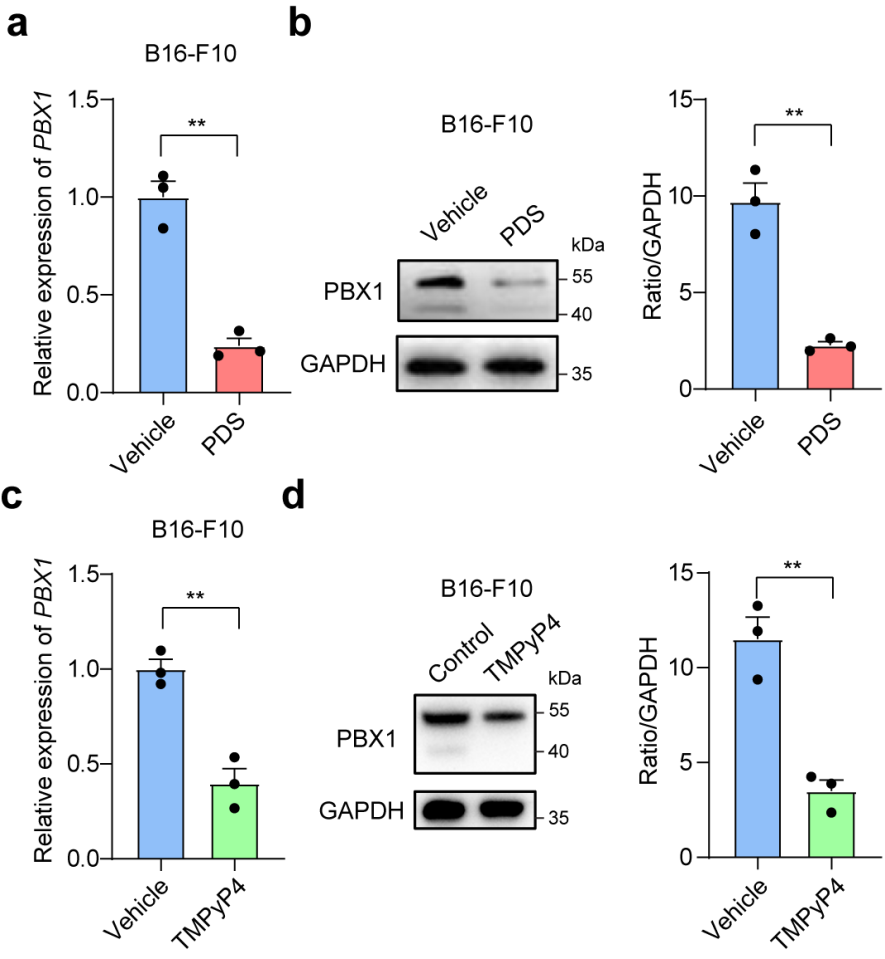


**Supplementary Fig. 6. PDS and TMPyP4 treatment inhibit PBX1 expression in B16-F10 cells. a-d** mRNA and protein levels of PBX1 in B16-F10 cells were detected by qRT-PCR and western blot. PDS (2 μM) or TMPyP4 (5 μM) treatment for 48 h inhibits the PBX1 mRNA (a,c) and protein levels (b, d. From left to right: PBX1 protein level, statistical analysis of western blot results. PBX1 protein levels were normalized to the GAPDH protein levels.). Data are shown as mean ± SEM of three independent experiments, two-tailed Student’s t test. ^**^*P* < 0.01.

**
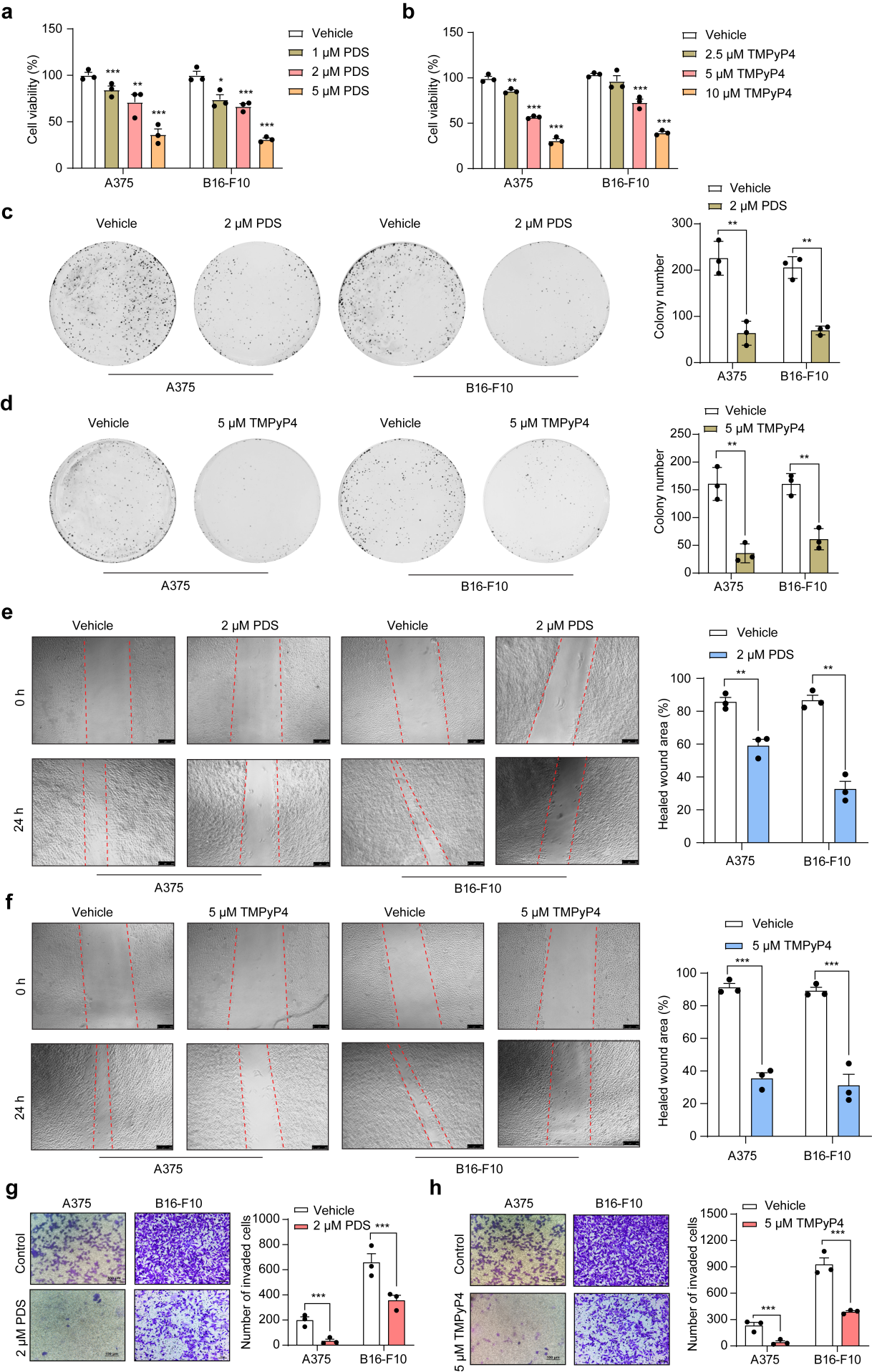
**

**Supplementary Fig. 7. PDS and TMPyP4 suppress melanoma progression *in vitro*.** **a,b** PDS (a) and TMPyP4 (b) treatment for 24 h reduce the cell viability of A375 and B16-F10 cells determined by CCK-8 assay. (**c-h)** The effects of PDS and TMPyP4 treatment for 24 h on cells plate colony formation (c-d. From left to right: colony formation assay, statistical analysis of colony number.), migration (e-f. From left to right: scratch-wound assay, statistical analysis of healed wound area.) and invasion (g-h. From left to right: Transwell assay, statistical analysis of invaded cells.) in A375 and B16-F10 cells. Data are shown as mean ± SEM of three independent experiments, two-tailed Student’s t test. ^**^*P* < 0.01, ^***^*P* < 0.001.

**
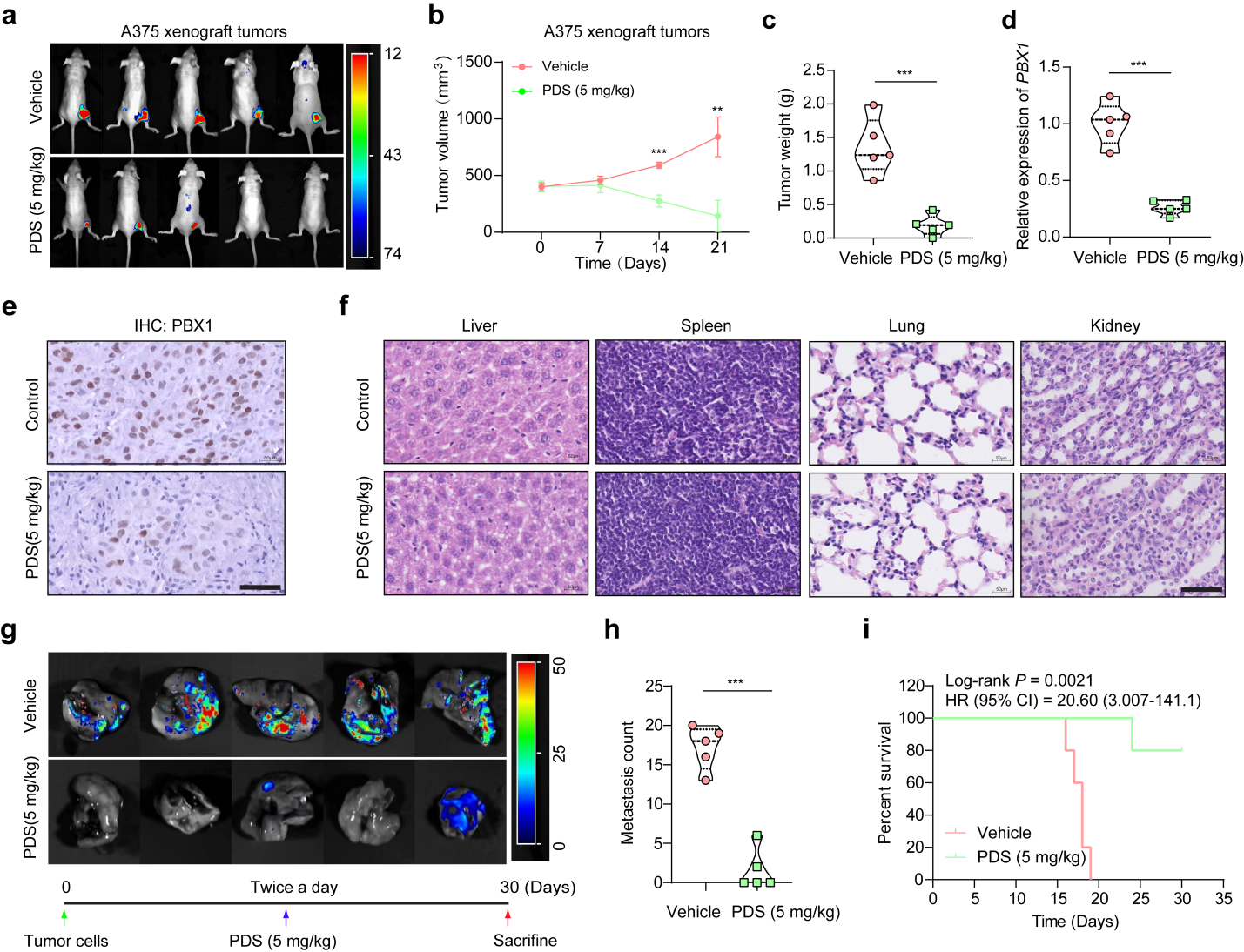
**

**Supplementary Fig. 8. PDS suppress melanoma progression *in* *vivo*.** **a** Representative bioluminescent (BLI) images (n = 5 mice/group) of A375 cells-derived xenograft tumor. **b** A375 cells-derived xenograft tumor volume measurements at day 0, 7, 14 and 21. **c** A375 cells-derived xenograft tumor weight of mice neoplasm after mice sacrifice. **d,e** The mRNA and protein levels of PBX1 were performed by qRT-PCR (d) and immunohistochemistry (IHC) staining (e) in A375 cells-derived xenograft tumor. Scale bar, 100 μm. **f** Representative Hematoxylin and eosin (H&E) staining of liver, spleen, lung and kidney from A375 cells-derived xenograft mice with PDS treatment (n=5 mice/group, three micrographs per individual were performed for the photomicrographs.). Scale bars, 100 µm. **g** Representative BLI images of lung tissues from each experimental group. **h** Violin plot showing BLI quantification of lung metastasis burden. **i** Mice were censored upon becoming moribund and followed for Kaplan-Meier survival curve analysis. n = 5 mice/group, Cox’s proportional hazards model, two-sided. ^**^*P* < 0.01, ^***^*P* < 0.001.

**
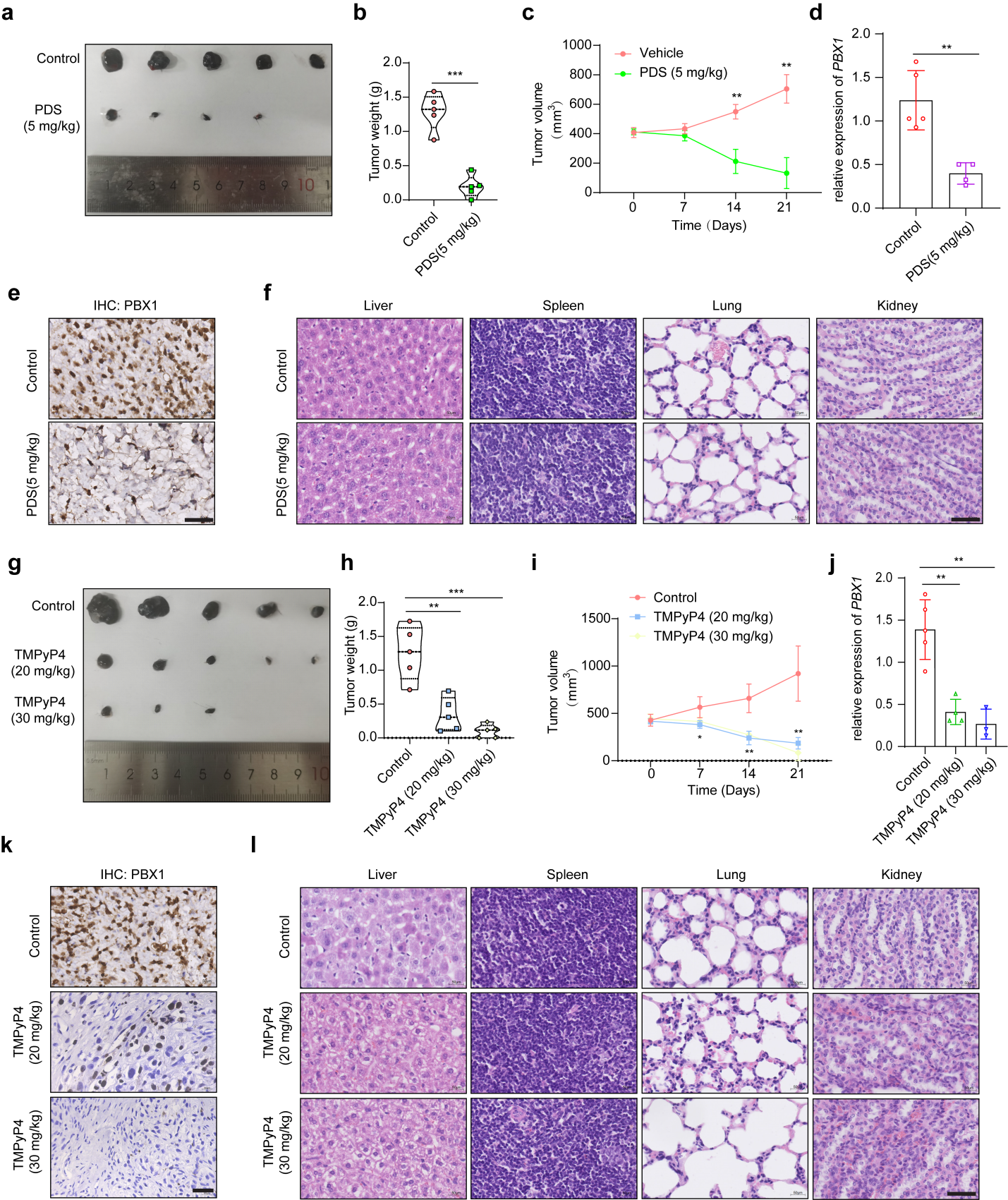
**

**Supplementary Fig. 9. PDS and TMPyP4 suppress** **B16-F10 cells growth *in* *vivo*.** **a** Representative tumor size (n=5 mice/group, left) and quantification of tumor weight (right) of isolated tumors from mice injected with B16-F10 cells. **b** Effects of PDS treatment in B16-F10 cells on subcutaneous tumor growth. **c** Tumor volumes were measured every seven days. The mice were killed 32 days after injection, and the tumors were excised. **d,e** The mRNA and protein levels of PBX1 were detected by qRT-PCR (d) and IHC staining (e) in tumor with PDS treatment. Scale bar, 100 μm. **f** Representative H&E staining of liver, spleen, lung and kidney from the mice with PDS treatment (n=5 mice/group, three micrographs per individual were performed for the photomicrographs.). Scale bars, 100 µm. **g** Representative tumor size (n=5 mice/group, left) and quantification of tumor weight (right) from the mice with TMPyP4 treatment. **h** Effects of TMPyP4 treatment in B16-F10 cells on subcutaneous tumor growth. **i** Tumor volumes were measured every seven days. The mice were killed 32 days after injection, and the tumors were excised. **j,k** The mRNA and protein levels of PBX1 were detected by qRT-PCR (j) and IHC staining (k) in tumor with TMPyP4 treatment. Scale bar, 100 μm. **l** Representative H&E staining of liver, spleen, lung and kidney from the mice with TMPyP4 treatment (n=5 mice/group, three micrographs per individual were performed for the photomicrographs.). Scale bars, 100 µm. ^*^*P* < 0.05, ^**^*P* < 0.01, ^***^*P* < 0.001.

**
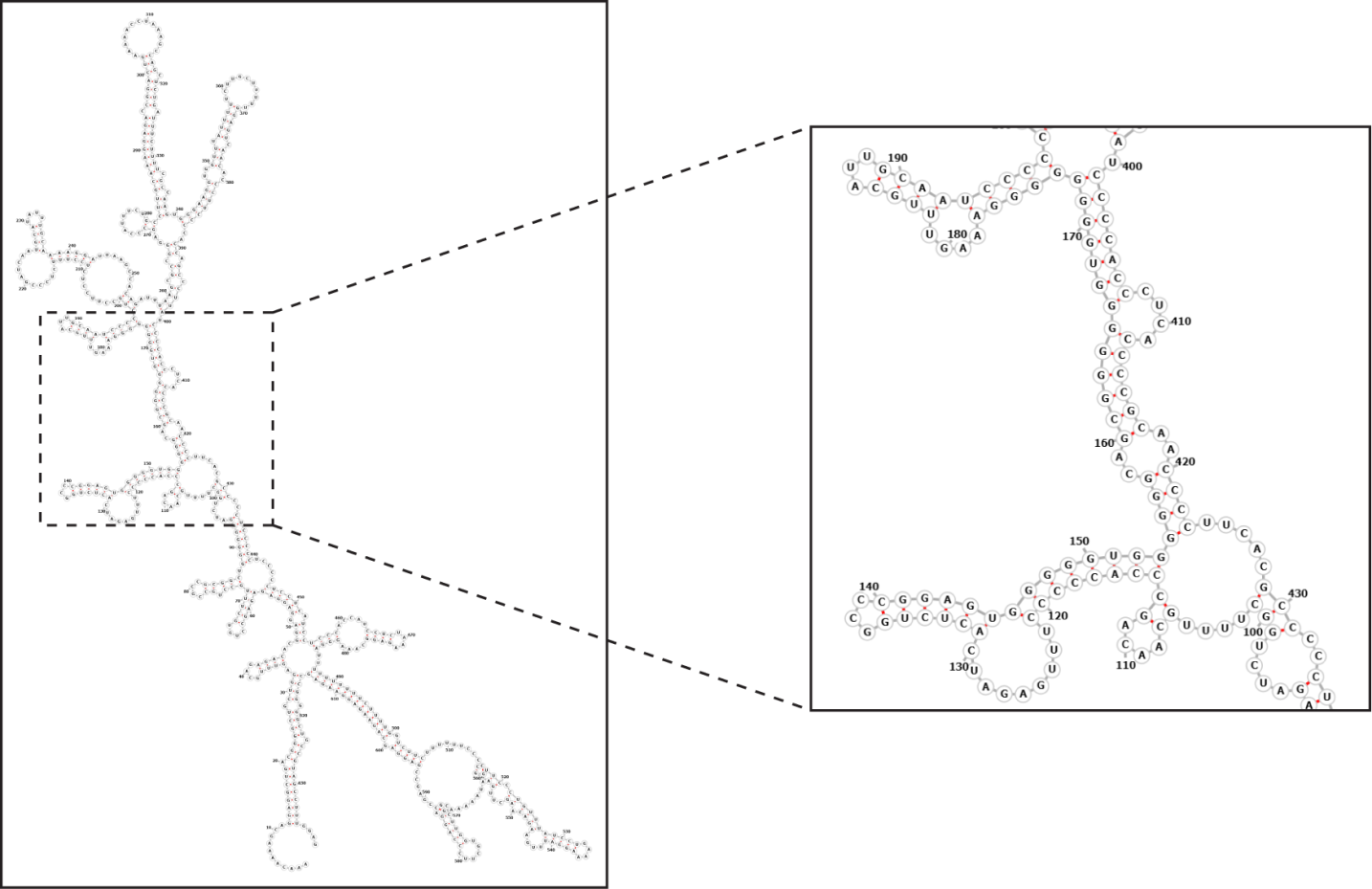
**

**Supplementary Fig. 10. Prediction of the RNA secondary structure of PBX1 5’UTR.**

**
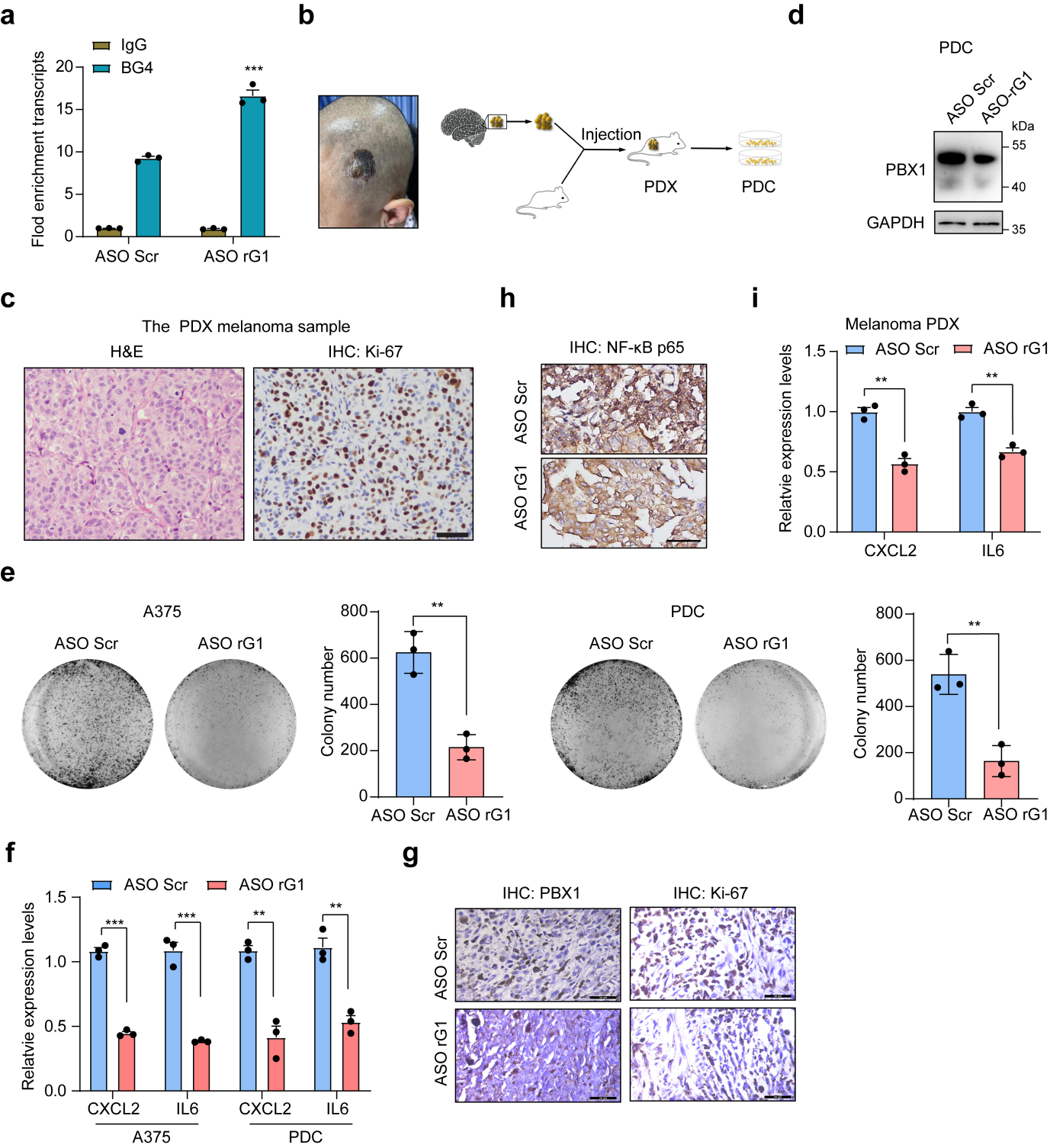
**

**Supplementary Fig. 11. PBX1 rG1 specific ASO inhibits melanoma progression.** **a** The occupancy of BG4 in the mRNA of PBX1 was measured by BG4 ChIP in A375 cells with ASO Scr or ASO treatment, followed by qRT-PCR. **b** Clinical sample for PDX (left, Clinical Sample 1) and schematic diagram of PDC model (right). **c** Representative H&E (left) and Ki67 IHC staining (right) of the melanoma tissues from patients. Scale bar: 100 μm. **d** The protein levels of PBX1 in PDC cells with ASO Scr or ASO rG1 (25 nM) treatment for 48 h. **e** Colony formation in A375 and PDC cells. From left to right: colony formation assay, statistical analysis of colony number. Data are shown as mean ± SEM of three independent experiments, two-tailed Student’s t test. **f** The expression levels of *CXCL2* and *IL6* in A375 and PDC cells without or with ASO rG1 treatment, detecting by qRT-PCR assays. Data are shown as mean ± SEM of three independent experiments, two-tailed Student’s t test. **g** The protein levels of PBX1 were performed by IHC staining. Scale bar: 50 μm. **h** Nuclear translocation of NF-κB p65 upon ASO rG1 treatment in PDX tumor tissues measured by IHC shows an increase in NF-κB translocation from cytoplasm to nucleus. Scale bar: 50 μm. **i** The expression levels of *CXCL2* and *IL6* in PDX tumor tissues, detecting by qRT-PCR assays. Data are shown as mean ± SEM of three independent experiments, two-tailed Student’s t test. ^**^*P* < 0.01, ^***^*P* < 0.001.

**
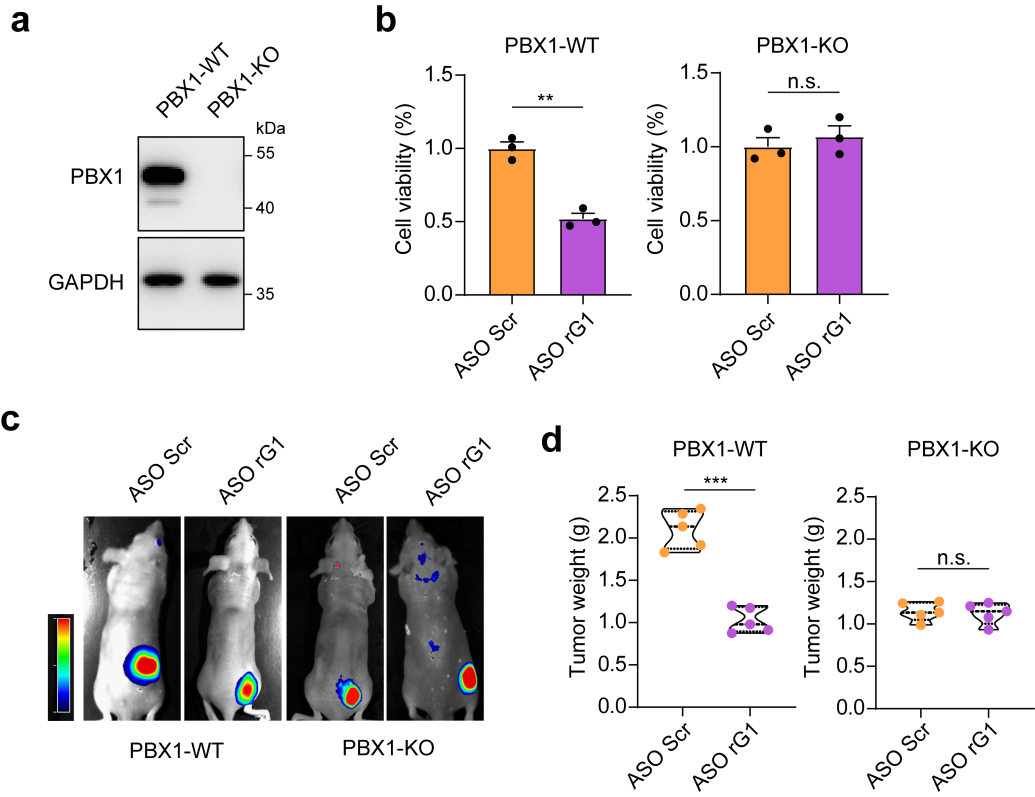
**

**Supplementary Fig. 12. Knockout of *PBX1* blocks the PBX1 rG1 specific ASO anti-melanoma effects. a** The protein expression of PBX1 in PBX1-wild type (WT) or PBX1-knockout (KO) A375 cells. **b** Cell proliferation in PBX1-WT or PBX1-KO A375 cells with ASO Scr or ASO rG1 (25 nM) treatment for 48 h. **c** Representative images of tumor with ASO Scr or ASO rG1 treatment (5 mice/group). **d** Tumor weight was measured after the mice sacrificed at day 35. Data are shown as mean ± SEM, two-tailed Student’s t test. n.s., not significant. ^**^*P* < 0.01, ^***^*P* < 0.001.

**
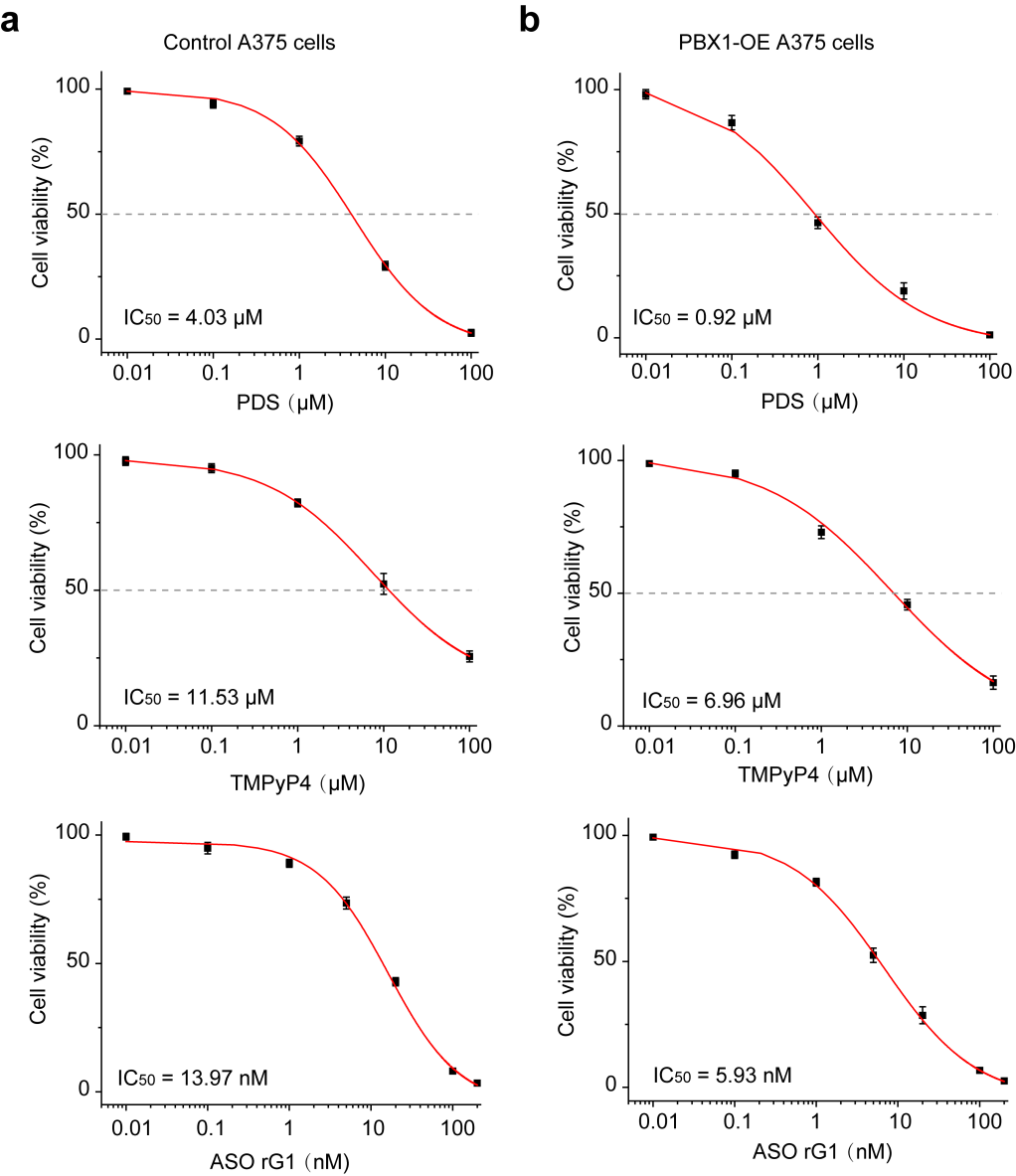
**

**Supplementary Fig. 13. PBX1-overexpressed melanoma cells were more sensitive to G4 ligands (PDS and TMPyP4) and ASO. a** The IC_50_ values of PDS, TMPyP4 and ASO rG1 for A375 cells without PBX1 overexpression, assessed by CCK-8 assay. **b** The IC_50_ values of PDS, TMPyP4 and ASO rG1 for A375 cells with PBX1 overexpression, assessed by CCK-8 assay. Data are shown as mean ± SEM of three independent experiments.


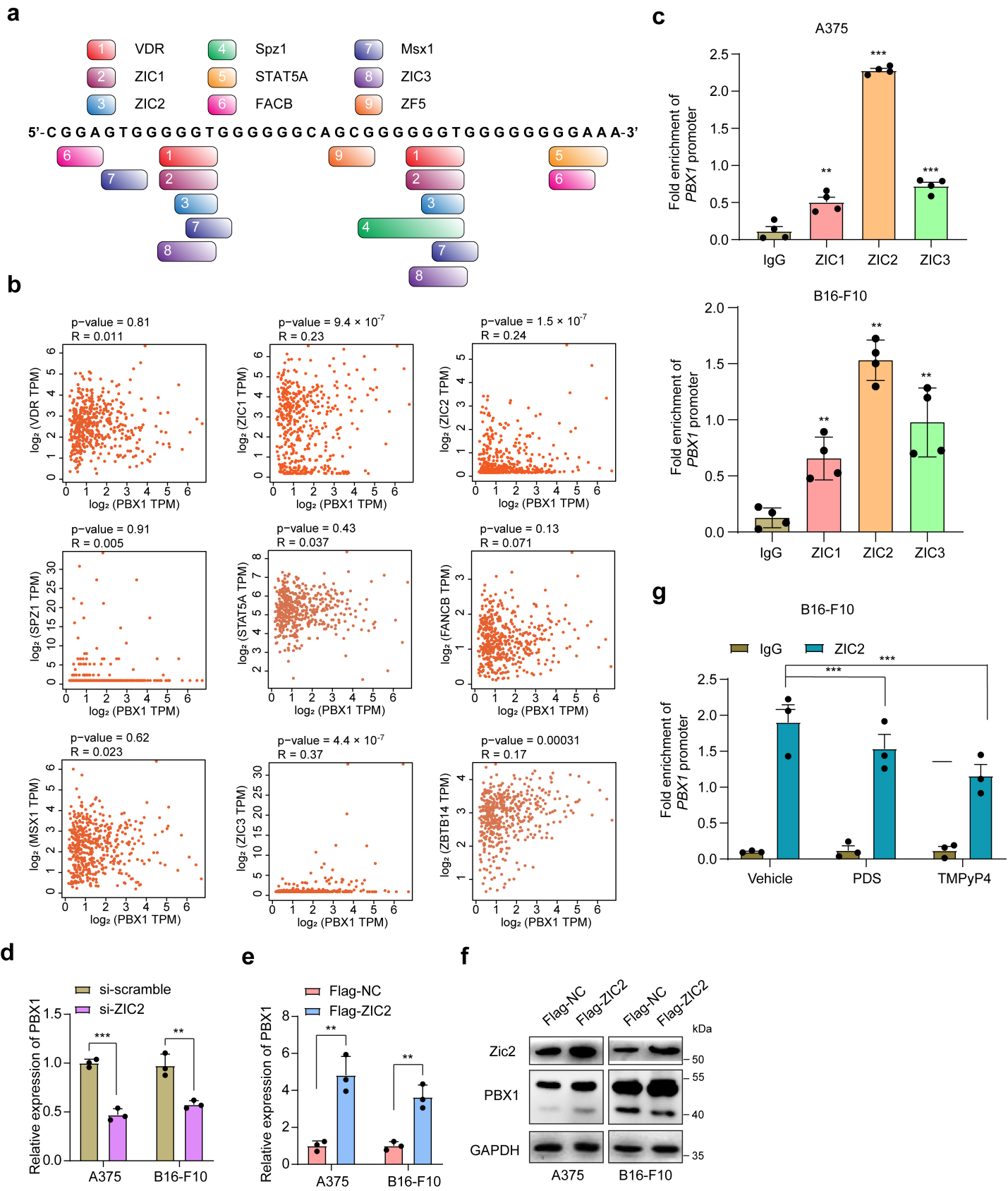


**Supplementary Fig. 14. Mechanisms of PBX1 G4s on regulating expression of PBX1. a** Prediction of transcription factors in PBX1 promoter region. **b** The correlation of PBX1 with the predicted 9 transcriptional factors in melanoma. **c** The occupancy of ZIC1, ZIC2 and ZIC3 in the promoter region of PBX1 was measured by ZIC1, ZIC2 and ZIC3 ChIP in A375 and B16-F10 cells, followed by qRT-PCR. **d** Relative expression levels of ZIC2 and PBX1 expressing si-scramble or si-*ZIC2*, detected by qRT-PCR. **e,f** Relative expression levels of ZIC2 and PBX1 expressing Flag-Ctrl or Flag-ZIC2, detected by qRT-PCR (e) and western blot (f). **g** The occupancy of ZIC2 in the promoter region of PBX1 was measured by ZIC2 ChIP in B16-F10 cells with or without 2 μM PDS and 5 μM TMPyP4 treatment for 48 h, followed by qRT-PCR. Data are shown as mean ± SEM of three independent experiments, two-tailed Student’s t test. ^**^*P* < 0.01, ^***^*P* < 0.001.
